# Enhancement of thermofluid characteristics via a triple-helical tube heat exchanger

**DOI:** 10.1038/s41598-025-89730-6

**Published:** 2025-02-26

**Authors:** Abdalla Gomaa, Yehia Gamal, Mahmoud M. Abdelmagied

**Affiliations:** Department of Refrigeration and Air Conditioning Technology, Faculty of Technology and Education, Helwan University, Cairo, 11282 Egypt

**Keywords:** Triple-helical tube, Annulus space, Curvature ratio, Inner annulus spacing, Engineering, Mechanical engineering

## Abstract

An investigation of the thermal and hydraulic performance of a novel triple-helical tube heat exchanger, the *THTHE,* is presented. The novel design is a modified design of a *DHTHE* created by adding a third tube to a *DHTHE* tube. The third passage is expected to enhance the thermal performance of the *DHTHE* as a result of an increase in the temperature gradient between the hot and cold fluids. The effects of the coil radius, inner annulus spacing, water inlet temperature, direction of flow arrangement, and Dean number were explored. Five test samples with different coil radii of 150 mm, 125 mm, and 90 mm and different inner annulus spacings of 6.2 mm, 9 mm, and 12 mm were examined. The test samples were designed, fabricated, and tested to demonstrate the influence of design parameters on the thermal and hydraulic performance of the triple-helical tube heat exchanger. The experimental runs were conducted on the hot water side with the water inlet temperature *T*_*h,i*_ ranging from 50:80 °C. Moreover, at Dean number 400 ≤ *De*_*h*_ ≤ 5500, corresponding to Reynolds number 2700 ≤ *Re*_*h*_ ≤ 31,000. Compared with the double-helical tube heat exchanger, the THTHE resulted in a higher Nusselt number by 146.1% and 109.3% for both the counterflow and parallel-flow arrangements, respectively. Furthermore, lowering the hot water source temperature from 80 to 50 °C results in a 60.6% increase in the Nusselt number of 60.6%, with no increase in the pumping power. Additionally, with a decreasing coil radius from 150 to 90 mm and inner annulus spacing from 12 to 6.2 mm, a significant increase in *Nu* occurs by 58.2% and 130.4%, respectively. A general correlation was presented for predicting *Nu*, *f*, and *ε*.

## Introduction

In recent years, global interest has focused on saving energy and increasing the efficiency of heat exchangers. Additionally, reducing the design size to occupy a small area will achieve a high heat transfer rate, thereby reducing the operating costs. The triple-helical tube heat exchanger (*THTHE)* was developed to meet these demands in all industries (food processing industries, refrigeration, air conditioning applications, power generation plants, chemical processes, and waste heat system recovery operations), with a high rate of heat transfer per unit volume. The *THTHE* is a modified design of the *DHTHE.* A modified version of this type was created to avoid double spirally coiled tube heat exchanger faults and achieve heat transfer enhancement. The design of the *THTHE* aims to increase the surface heat transfer area per unit length by creating a third fluid passage for the external surface of a double-helical tube heat exchanger (*DHTHE)*. The novel passage increases the side area (surface between the intermediate tube and the novel passage in the outer tube), and an increase in the heat transfer characteristics is expected. Another advantage of using a *THTHE* is that the generation of a centrifugal force and secondary flow due to flow in the helical passages improves the fluid turbulence and heat transfer rates from the heat exchangers.

Many studies have experimentally and numerically achieved the performance of straight and curved tubes by using either double or triple tubes. Hoshi^1^ presented an experimental study of *DHTHEs* using four coils. The results revealed an increase in the Nusselt number, *Nu,* with increasing curvature ratio. Sahoo et al.^2^ investigated triple tubes with different flow arrangements. The results showed that the countercurrent arrangement with a high flow rate of water has better efficiency than the other arrangements do. Gomaa et al.^3^ investigated triple-tube heat exchanger performance with inserted ribs. An increase in the convective heat transfer occurred when the inserted ribs were used. Rahman^4^ presented a comprehensive review of passive heat transfer enhancement methods in helical tube. The passive methods such as fins, inserts, geometry modifications, and baffles were evaluated. Abdelmagied^5^ Presented an experimental investigation of a triple helical tube with inner twisted tube heat exchanger. The results indicated that twisted tubes have greater *Nu* by 24.7% at the expense of increasing *f* by 36.4% and New correlations to predict the *Nu* and *f* were presented. Gaur et al.^6^ investigated the heat transfer and fluid flow in a triplex tube heat exchanger using water-based 1% *v*/*v* MXene and Al_2_O_3_ nanofluids. The investigation shows that using MXene nanofluid resulted in a 12.51% increase in heat transfer coefficients compared to Al_2_O_3_ nanofluid and a 20% increase compared to water in a counter-flow configuration. Touatit and Bougriou^7^ used FORTRAN code to achieve the optimal triple tube diameter. The technoeconomic method was used to optimize the heat exchanger and decrease the total cost.

Li et al.^8^ presented a numerical study on the flow and heat transfer characteristics. The results indicated that the friction pressure drop is affected mainly by the vapor quality, mass flow, and saturation pressure, whereas the mass does not affect them. Correlations for estimating heat transfer characteristics were presented by Gomaa et al.^9^ for a double helical tube in a tube heat exchanger. Radulescu et al.^10^ presented an experimental investigation for a heat transfer coefficient solver for triple tubes in the transition regime, where a useful correlation of the partial heat transfer coefficient was obtained. Tamkhade et al.^11^ experimentally analyzed the use of triple tubes for cooling oil. The change in oil temperature is analyzed under various operating conditions. Lubis et al.^12^ presented a study of triple tubes with a counter flow design. The hot fluid flows in the inner annulus space, whereas the cold fluid flows in the inner tube and the outer annulus, with the hot water entering at 60 °C and the cold air entering at 25 °C. Abdelmagied^13–15^ presented a numerical 3-D model and experimental procedure to study the thermal and hydraulic properties in light of different operating and design parameters for different triple curved coiled tubes. Experiments were performed in turbulent fluid flow, and new correlations to predict *Nu*_*h*_*, f*_*h*_ and *ε* were presented.

Quadir et al.^16^ studied the performance of a triple tube for two different flow types. The change in the temperature of the three liquids along the length of the heat exchanger for different flow rates is considerably different for the two arrangements.

Abdelmagied^17^ presented an experimental and numerical the thermal performance of a new triple helical tube with inner twisted tube. The results show that a significant increases in *Nu* compared to a double helical tube with inner twisted tube, while having a negligible effect on the friction factor (*f*_*h*_).

Gaur et al.^18^ examined the impact of hybrid nanofluids with various nanoparticle shapes (MWCNT, Al2O3, Graphene, and Fe3O4) in a triple concentric tube exchanger. The effect of hot water and hybrid nanofluids on heat transfer efficiency through energetic and energetic performance were analysis. Rahman et al.^19^ reviews the continuous and discrete swirl-inducing techniques for enhancing heat transfer through geometry alterations or flow deflectors. The results showed that all angles swirl-inducing devices improved fluid mixing, reduced thermal boundary layers, and created turbulent eddies.

Memon et al.^20^ presented a study on the modeling and simulation of a triple tube using different materials. The results show that mild steel was the best of the seven materials tested. *Triple* concentric-tube latent heat thermal energy storage was evaluated by BaŞal and Ünal^21^. The results indicated that the most important design parameters are the radial location and thickness of the phase change material that the annulus fills. Zhao et al.^22^ presented a 3D model of a triple-pipe helical system to investigate the melting characteristics of phase change material. The results showed that the secondary flows within the inner pipe are more effective in enhancing melting than those in the outer pipe. Gómez et al.^23^ presented a theoretical model to calculate the thermal parameters of a triple-tube heat exchanger, using a formulation similar to that for double-tube heat exchangers. The theoretical model shows an average errors of 7% and 12%, while the CFD simulation shows errors of 5% and 10% for straight and U-shaped designs, respectively.

Rico et al.^24^ studied the characterization of triple tubes with corrugated tubes via artificial neural networks. There is good agreement with the experimental data, with deviations of less than 1.91% and 3.82% for the heat transfer coefficient and pressure drop, respectively. Ahamad and Verma^25^ presented an experimentally investigated the thermal modeling and performance of three-fluid heat exchangers, with an enhanced version of the double-pipe heat exchanger. The results showed that the increasing of the flow rate improves the heat transfer coefficient, while effectiveness varies with residence time and capacity ratio.

The results suggested that the use of the smaller rib height and lower rib pitch with the highest nanoparticle concentration is greater than that of the other cases. Kumar and Chandra^26^ presented an experimental investigation of heat transfer in a triple-tube heat exchanger with coiled spring inserts. ​The results showed that the thermal performance and effectiveness increased as the pitch of the spring insert decreased. Singh et al.^27^ investigated the thermohydraulic behavior of triple tubes at all possible flow arrangements at 2800 ≤ Re ≤ 11,000 while hot water was in the inner annulus. Rahman and Hasnain^28^ reviewing the improvements in the thermal efficiency of heat exchangers using passive enhancement techniques, such as twisted tape, wire coil, and swirl flow generators. A recent research trend is using nanofluids with techniques like fins and twisted tape inserts to enhance heat transfer and reduce exergy losses in heat exchangers. Hameed and Hamad^29^ evaluated a modified heat exchanger performance through both experimental and mathematical analyses. The triangular fins were added to the external surface of a longitudinal helical heat exchanger. Adding the fins improved the helical heat exchanger, boosting thermal performance by over 10%, effectiveness by 11%, and the *Nu* by 16.5%.

Although a significant amount of research has been performed on *DHTHEs*, few studies have focused on the thermal and hydraulic performance of curved *THTHEs*. Therefore, this study experimentally presents the thermohydraulic performance of a novel design of a *THTHE*. To achieve the optimum operating conditions and design parameters, the effects of the coil radius, inner annulus spacing, water inlet temperature, and flow arrangement were covered and compared with those of the *DHTHE* as a particular reference.

## Experimental test rig

**Fig. 1:**
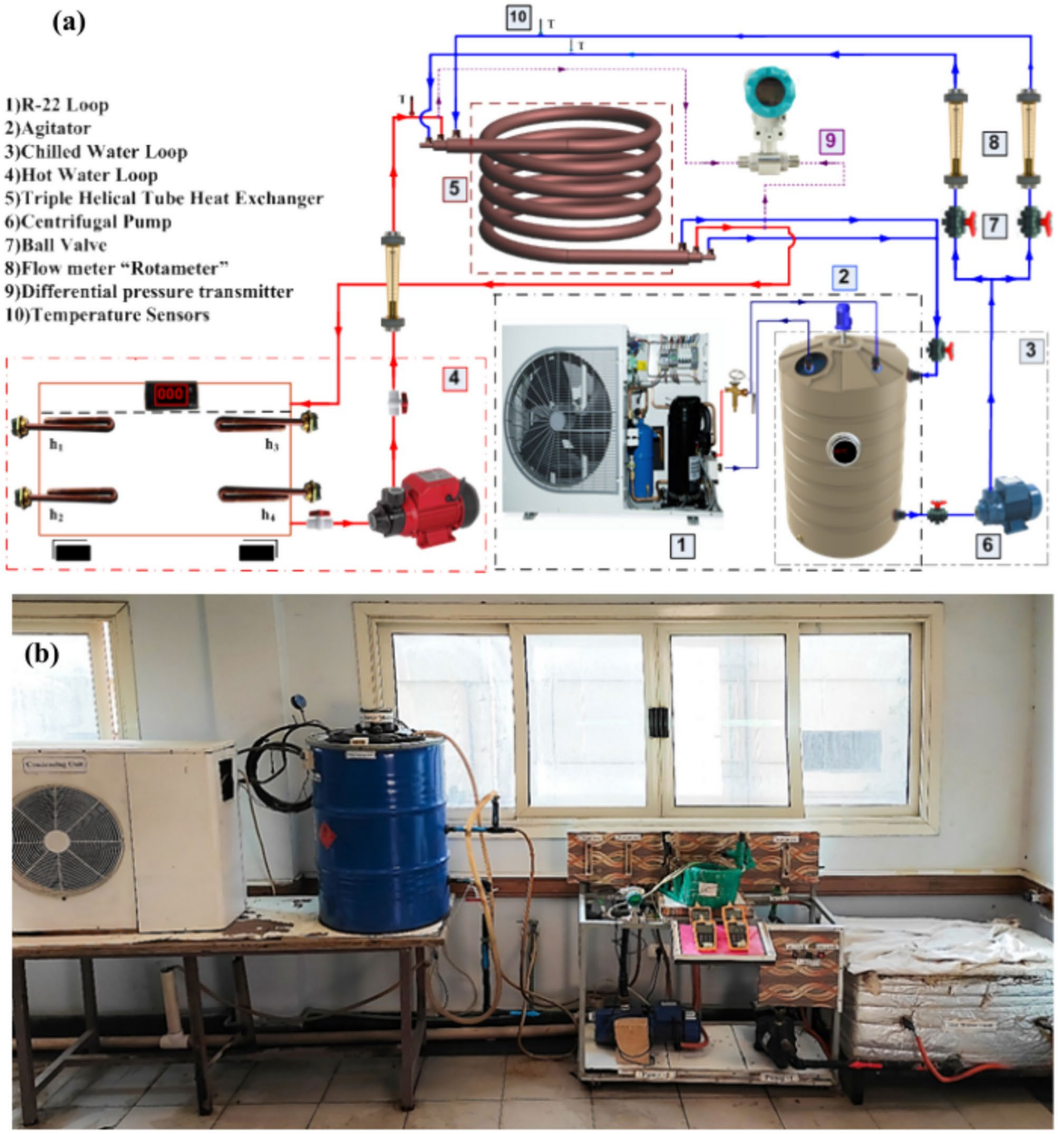
The experimental device, (**a**) Schematic diagram and (**b**) Photograph view.

**Fig. 2:**
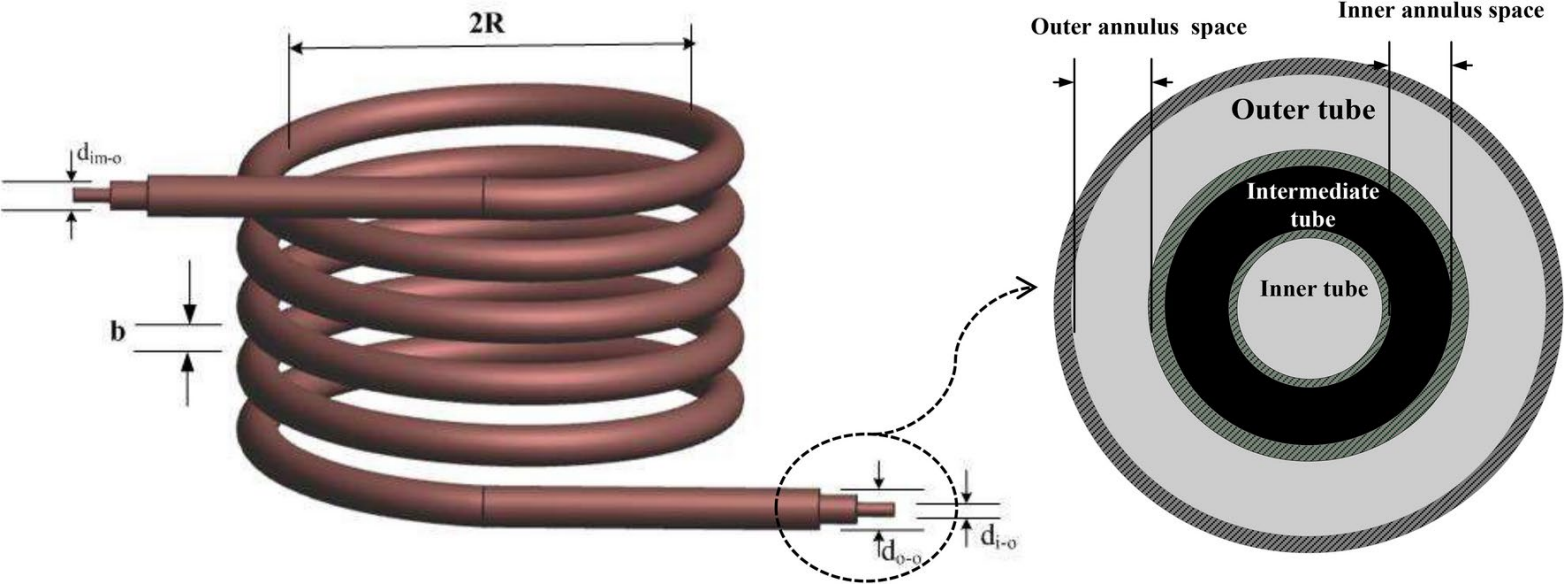
Geometry of a *THTHE*.

**Fig. 3:**
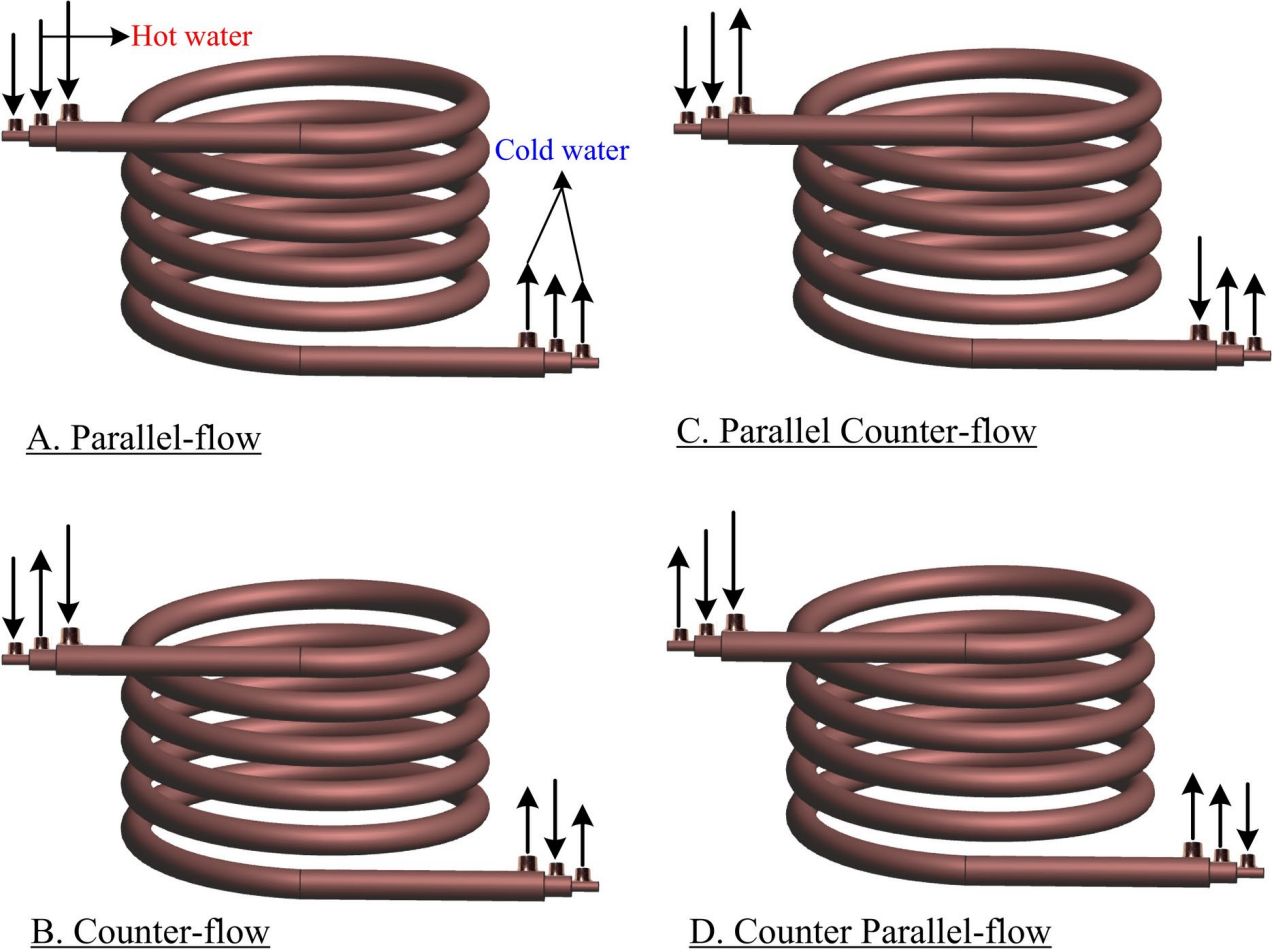
Flow patterns of a *THTHE*.

Figure 1a and b display the *THTHE* test rig, which consists of a closed water cooling circuit, a closed hot water circuit, and a *THTHE* circuit. The closed-circuit water cooling components are a cooling circuit of 3.7 kW total capacity, an insulated tank of 0.15 m^3^ and adjustable temperature control. The closed hot water circuit components are an insulated 0.25 m^3^ tank, a temperature control adjustable device, and electric heaters with a total capacity of 6 kW. The *THTHE* loop consists of three copper tubes (*ρ* = 8978 kg/m^3^*, C* = 381 J/kg). K and* k* = 387.6 W/m.K). The coil is helically wrapped at a constant diameter (Fig. 2), where *R and b* are the *THTHE* radius and pitch, respectively. The geometrical dimensions are summarized in Table 1. The hot water was fed to the *THTHE* via a 1 hp centrifugal pump through the intermediate tube, and the flow rate was determined by a ball valve and a rotameter from 0.016 to 0.3 kg/s. The hot water is supplied at temperatures ranging from 50 to 80 °C. In addition, cold water is pumped at 20 °C ± 0.5 °C in the *THTHE* inner tube and outer annulus (*outer tube*) via a centrifugal pump with a capacity of 2 hp. The flow rates were determined by ball valves and two parameters from 0.016–0.3 kg/s. The outer side area of the *THTHE* is thermally insulated. *THTHE* is classified according to the flow pattern into four possible patterns, namely, counter, parallel, counterparallel, and parallel-counter patterns, as shown in Fig. 3^3^ The temperatures of the *THTHE* inlet and outlet fluids were measured via six precalibrated K-type thermocouples (± 0.1 °C accuracy). The pressure drop across the *THTHE* was recorded by a precalibrated digital differential pressure transmitter (± 0.5 kPa accuracy). In the present work, 4800 runs (3200 runs for the *THTHE* and 1600 runs for the *DHTHE*) were carried out to obtain the results. The steady-state condition was achieved by allowing the system to observe the stability of the measuring parameters (temperatures and pressure drop) for approximately 40 min.

**Table 1:** The detailed parameters of *THTHE*.

Test specimen	*d*_*i,o*_ (mm)	*d*_*im,o*_ (mm)	*do,o* (mm)	(mm)	*L *(mm)	$d_{{hy}} = d_{{im,i}} - d_{{i,o}}$(mm)	$\delta = d_{{hy}} /2R$	*b* (mm)	*N*
*A*	7.92	15.9	22.2	150	4430	6.2	0.020	29.5	4.20
*B*	7.92	15.9	22.2	125	4080	6.2	0.024	29.5	5
*C*	7.92	15.9	22.2	90	4120	6.2	0.034	29.5	6.55
*D*	7.92	19	28.6	90	4230	9.38	0.052	29.5	6.83
*E*	7.92	22.2	28.6	90	4320	11.28	0.062	29.5	6.95

## Data reduction

The thermal and hydraulic performance of the *THTHE* are described and summarized via the following equations:

The heat transfer from the hot water to the cold water in the inner tube and outer annulus sides can be calculated as follows^3^:

1 $$\mathop Q_h\limits^{ \cdot } =\mathop m_h\limits^{ \cdot } C_{h} (T_{{h_{i} }} - T_{{h_{o} }} )$$

2 $$\mathop Q_{c_1}\limits^{ \cdot } = m_{c_1} C_{c} (T_{{c_{1} ,o}} - T_{{c_{1} ,i}} )$$

3 $$\mathop Q\limits^{ \cdot }_{c_2} = m_{c_2} C_{c} (T_{{c_{2} ,o}} - T_{{c_{2} ,i}} )$$

The heat balance between the *THTHE* cold and hot water was determined as:

4 $$\mathop Q_h\limits^{ \cdot } = (\mathop Q\limits^{ \cdot }_{{c_{1} }} + \mathop Q\limits^{ \cdot }_{{c_{2} }} )$$

The average heat transfer rate of *the THTHE* between the three fluids was determined by Gomaa et al.^3^:

5 $$\mathop Q\limits^{ \cdot }_{avg} = \frac{{\mathop Q_h\limits^{ \cdot } + \mathop Q\limits^{ \cdot }_{1,2} }}{2}$$

where $\mathop Q\limits^{.}_{{c_{1,2} }}$ is the aggregate of the cold-water heat transfer rate:

6 $$\mathop Q\limits^{ \cdot }_{{c_{1,2} }} = (\mathop Q\limits^{ \cdot }_{{c_{1} }} + \mathop Q\limits^{ \cdot }_{{c_{2} }} )$$

The convective heat transfer coefficient for the inner annulus side, *h*_*im*_*,* can be calculated as^3^:

7 $$\mathop Q_h\limits^{ \cdot } = h_{im} \;A\;LMTD_{avg}$$

8 $$h_{im} = \frac{{\mathop Q\limits^{ \cdot }_{h} }}{{A_{{}} LMTD_{avg} }}$$

The *THTHE* average logarithmic-mean temperature difference *LMTD*_*avg*_ between the three fluids can be calculated as^3^:

9 $$LMTD_{avg} = \frac{{LMTD_{hc1} + LMTD_{hc2} }}{2}$$

**Fig.4:**
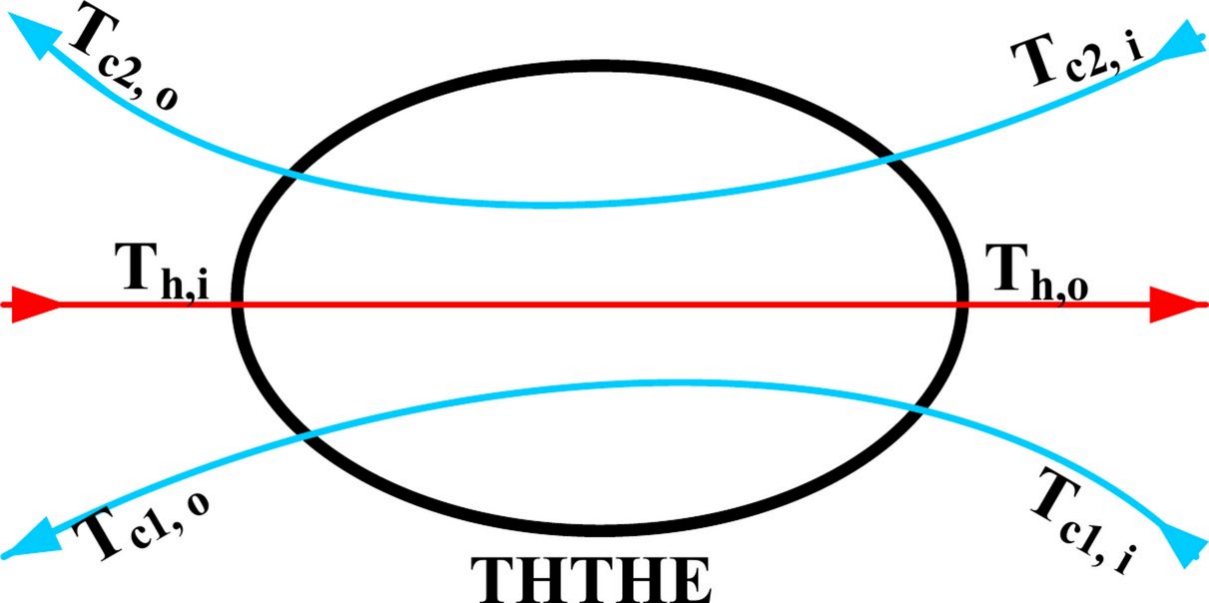
Estimated method for calculating LMTD.

*LMTD*_*hc1*_ is between hot and cold water in the inner tube, whereas *LMTD*_*hc2*_ is between hot and cold water in the outer annulus side (Fig. 4).

10 $$LMTD_{hc1} = \frac{{\Delta T_{hc1.1} + \Delta T_{hc1.2} }}{{\ln \left( {\frac{{\Delta T_{hc1.1} }}{{\Delta T_{hc1.2} }}} \right)}}$$

11 $$LMTD_{hc2} = \frac{{\Delta T_{hc2.1} + \Delta T_{hc2.2} }}{{\ln \left( {\frac{{\Delta T_{hc2.1} }}{{\Delta T_{hc2.2} }}} \right)}}$$

where

$$\Delta T_{hc1.2} = \, \left( {{\text{T}}_{{{\text{h}},{\text{i}}}} {-}{\text{T}}_{{{\text{c1}},{\text{o}}}} } \right),\Delta T_{hc1.1} = \, \left( {{\text{T}}_{{{\text{h}},{\text{o}}}} {-}{\text{T}}_{{{\text{c1}},{\text{i}}}} } \right),\Delta T_{hc2.2} = \, \left( {{\text{T}}_{{{\text{h}},{\text{i}}}} {-}{\text{T}}_{{{\text{c2}},{\text{o}}}} } \right),{\text{ and }}\Delta T_{hc2.1} = \, \left( {{\text{T}}_{{{\text{h}},{\text{o}}}} {-}{\text{T}}_{{{\text{c2}},{\text{ i}}}} } \right)$$

The Reynolds number and Nusselt number for the inner annulus side are determined as:

12 $${\text{Re}}_{h} = \frac{{\rho_{{}} v_{h} d_{hy} }}{\mu }$$

13 $$Nu_{h} = \frac{{h_{{}} d_{hy} }}{k}$$

where *d*_*hy*_ is defined as:

14 $$\begin{gathered} d_{hy} = \frac{{4A_{annulus} }}{{\pi (d_{im,i} + d{}_{i,o})}} \hfill \\ d_{hy} = 4 \cdot \frac{{\left[ {\pi /4} \right]({d}^{2}_{im,i} - {d}^{2}_{i,o} )}}{{\pi (d_{im,i} + d{}_{i,o})}} = \frac{{(d_{im,i} - d_{i,o} )(d_{im,i} + d_{i,o} )}}{{(d_{im,i} + d{}_{i,o})}} \hfill \\ d_{hy} = (d_{im,i} - d{}_{i,o}) \hfill \\ \end{gathered}$$

The *THTHE* Darcy‒Weisbach friction factor is calculated as follows:

15 $$f_{h} = \frac{{2_{{}} \Delta P_{{h_{{}} }} d_{hy} }}{{\rho_{{}} L_{{}} v_{h}^{2} }}$$

The effectiveness of the *THTHE* is calculated as:

16 $$\varepsilon = \frac{{\mathop Q\limits^{ \cdot }_{avg} }}{{\mathop Q\limits^{ \cdot }_{\max } }} = \frac{{\mathop Q\limits^{ \cdot }_{avg} }}{{(\mathop m\limits^{ \cdot } C)_{\min } (T_{{h_{i} }} - T_{{c_{i} }} )}}$$

The heat transfer per unit pumping power of the *THTHE* is calculated as^12^:

17 $$\mathop Q\limits^{ \cdot } /PP = \frac{{\mathop m\limits^{ \cdot }_{h} c_{h} \Delta T{}_{h}}}{{(\mathop m\limits^{ \cdot }_{h} \Delta p_{h} /\rho_{h} ) + (\mathop m\limits^{ \cdot }_{{c_{2} }} \Delta p_{{c_{2} }} /\rho_{{c_{2} }} )}}$$

The *THTHE* performance index (*η*) for the *THTHE* based on the double-helical tube is calculated as:

18 $$\eta = {{\left( {\frac{{Nu_{THTHE} }}{{Nu_{DHTHE} }}} \right)} \mathord{\left/ {\vphantom {{\left( {\frac{{Nu_{THTHE} }}{{Nu_{DHTHE} }}} \right)} {\left( {\frac{{f_{DHTHE} }}{{f_{DHTHE} }}} \right)}}} \right. \kern-0pt} {\left( {\frac{{f_{DHTHE} }}{{f_{DHTHE} }}} \right)}}^{1/3}$$

## Measurements uncertainty

The uncertainty of the experimental error should be considered when implementing the error in the results. A differential approximation method was applied for error analysis, and the results of the uncertainty measurements are given in Table 2.

**Table 2:** Experimental accuracy and uncertainty of measured parameters.

Device/parameter	Range	Accuracy (%)	Uncertainty (%)
Flow rotameters, kg/s	0.016–0.3	± 0.5	3.3
K-type Thermocouples, oC	− 200 to 1200	± 0.1	0.12
Differential pressure digital transmitter, kPa	0.5–500	± 0.5	± 0.59
Reynolds number	2700–31,000	–	± 3.36
*f*_*h*_	–	–	± 3.41
*Nu*_*h*_	–	–	± 5.22
$\varepsilon$	–	–	± 3.51
*Q*./*PP*	–	–	± 3.54

## Results and discussion

**Fig. 5:**
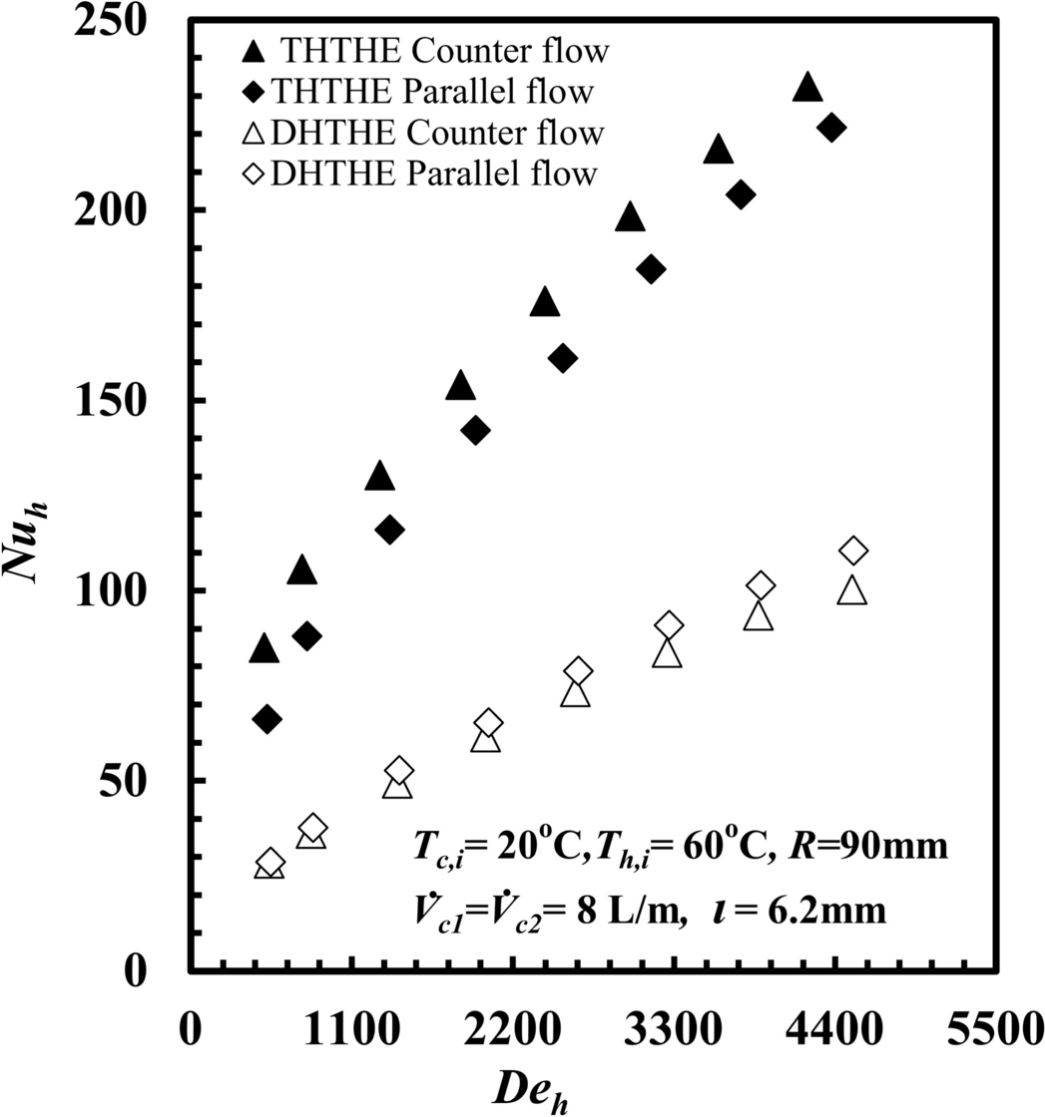
*Nu*_*h*_ versus *De*_*h*_ for *DHTHE* and *THCTHE*.

An experimental comparison was carried out between the *THTHE* and *DHTHE*. The main objective is to provide a precise view of the thermal and hydraulic performance of the *THTHE* as a modified version of the *DHTHE*. The comparison results are displayed for test sample C with respect to *T*_*h,*__i_ = 60 °C, counterparallel flow, and a cold-water flow rate of 8 L/s for both the inner and outer tubes. Figure 5 displays *Nu*_*h*_ versus *De*_*h*_ for both the *THTHE* and *DHTHE* for the bath counter and parallel flow patterns. The figure shows that *Nu*_*h*_ increases with *De*_*h*_ for all the experiments. Furthermore, the *THTHE* Nusselt number*, Nu*_*h,*_ is greater than the *DHTHE* for both counterflow and parallel flow. At the same *De*_*h*_ = 3300, the *THTHE, Nu*_*h*_ is greater than *DHTHE* by 146.1% and 109.3% for both counter and parallel patterns, respectively. This is due to an increase in the heat transfer rate of the THTHE compared with that of the *DHTHE* as a result of an increase in the surface contact area due to the addition of a new flow passage. The new path of the fluid also leads to an increase in the temperature gradient of the hot water in the *THTHE*, which occurs at a cross-section other than that of the *DHTHE*; this increases the heat transfer coefficient, and consequently, *Nu*_*h*_ increases.

**Fig. 6:**
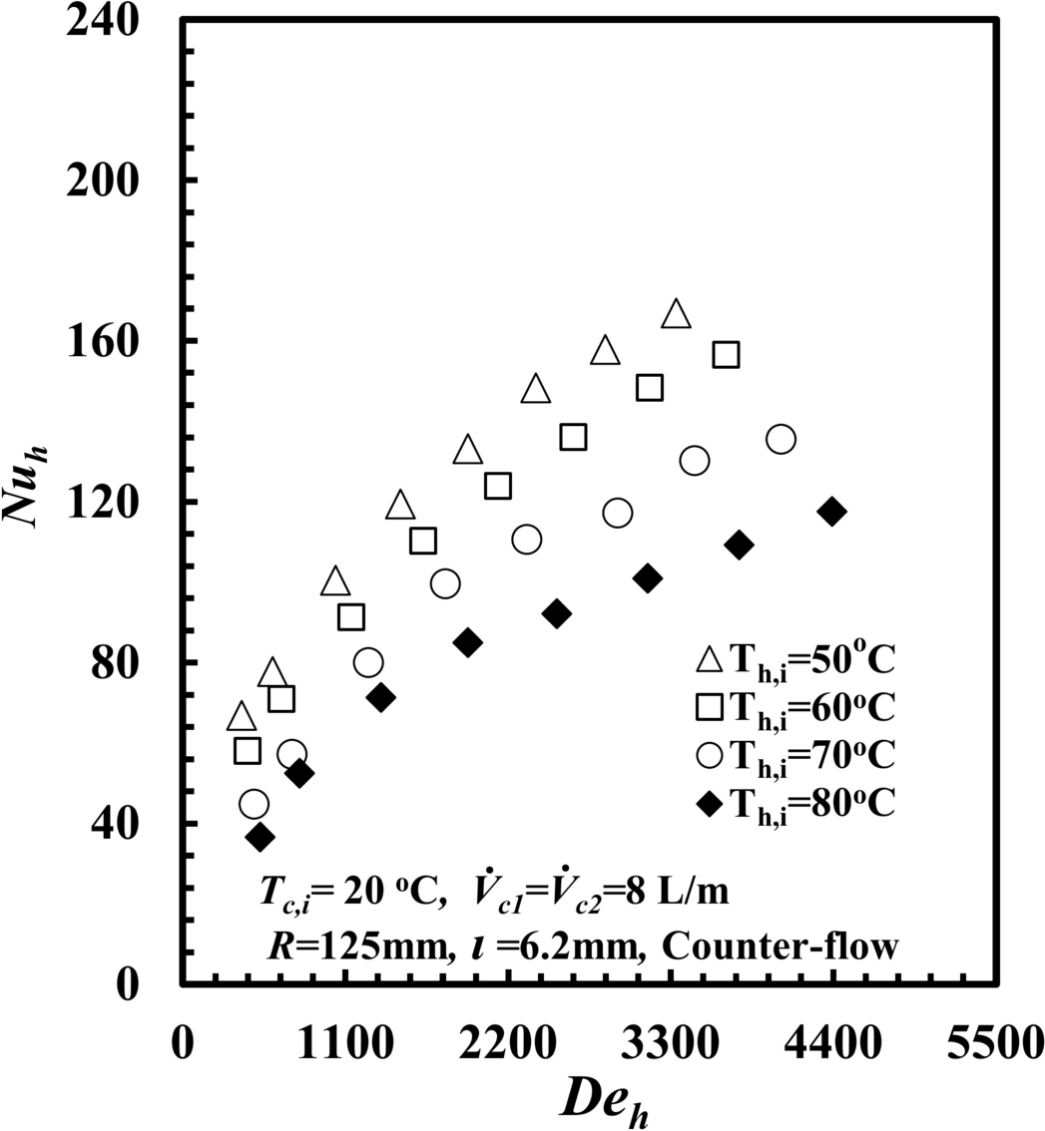
*Nu*_*h*_ versus *De*_*h*_ with different temperature for *THTHE*.

Figure 6 explains the influence of supply *T*_*h,i*_ on *Nu*_*h*_ against *De*_*h*_. The figure shows that *Nu*_*h*_ improved with increasing *De*_*h*_ for all the experiments. At *De*_*h*_ = 3300, the *Nu*_*h*_ for *T*_*h,i*_ = 50°C is greater than by approximately 10.5%, 30.5%, and 60.6% compared with *T*_*h,i*_ = 60°C, 70°C, and 80°C, respectively. According to Eq. (10), when *T*_*h,i*_ decreases from 80 to 50°C, both the heat transfer rate and *∆T*_*LMTD*_ decrease together, but the decrease in *∆T*_*LMTD*_ is relatively greater than the heat transfer rate. This caused an increase in both *h*_*im*_ and *Nu*_*h*_ consequently.

**Fig. 7:**
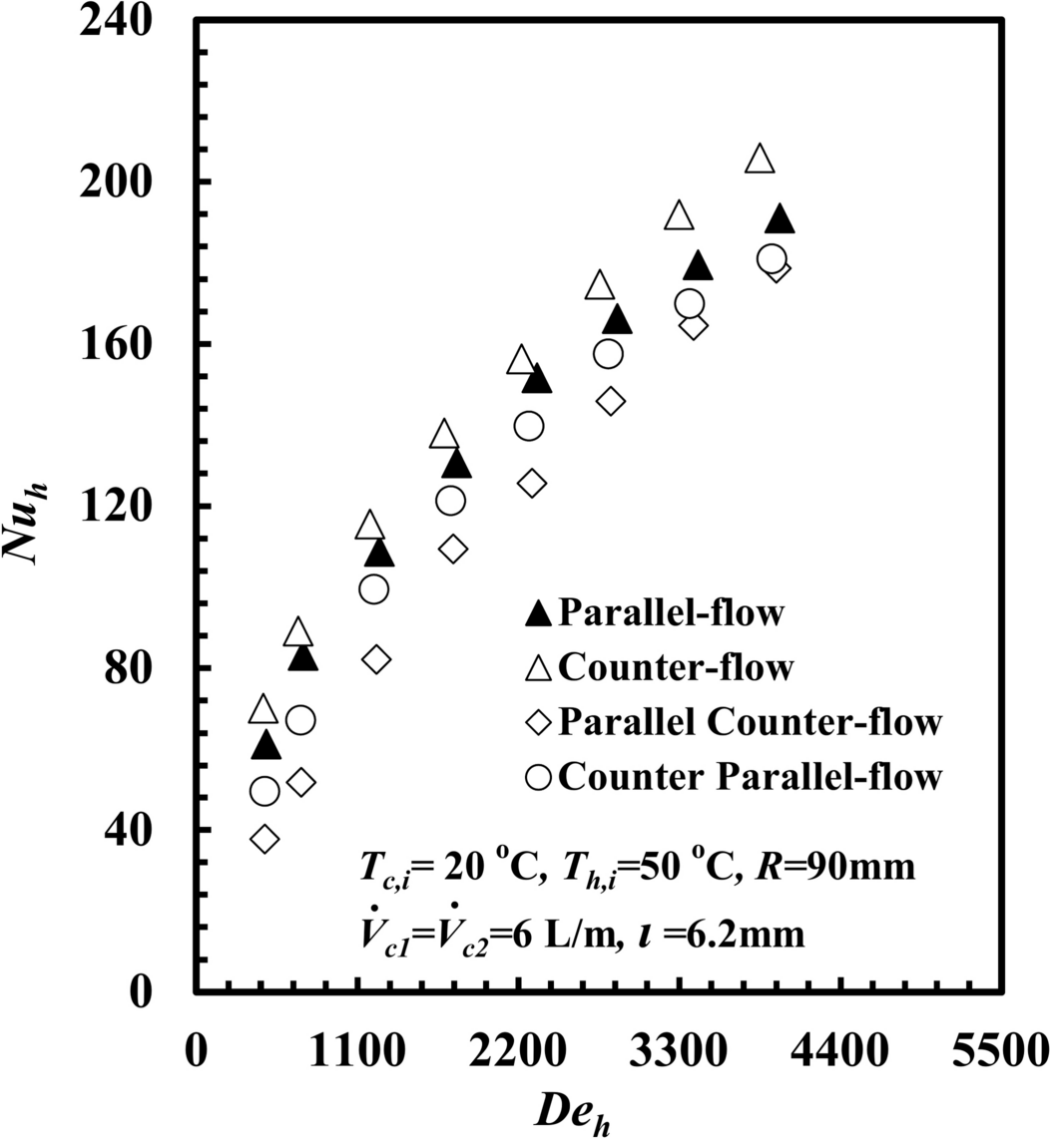
*Nu*_*h*_ versus *De*_*h*_ at different *THTHE* flow arrangements.

The relationship between *THTHE Nu*_*h*_ and *De*_*h*_ is shown in Fig. 7 for different patterns of water flow. The *Nu*_*h*_ increased in counter flow patterns compared with parallel, counter-parallel, and parallel-counter flow patterns by approximately 8.9%, 14.2%, and 20%, respectively, at the same value of *De*_*h*_ = 3300. This can be referred to as increasing *∆T*_*h*_ as well as *∆T*_*LMTD*_ between hot and cold fluids in the counter flow pattern compared with the three other patterns. This reflects increasing $\dot{Q}$ and consequently *Nu*_*h*_.

### Effect of the coil radius

**Fig. 8:**
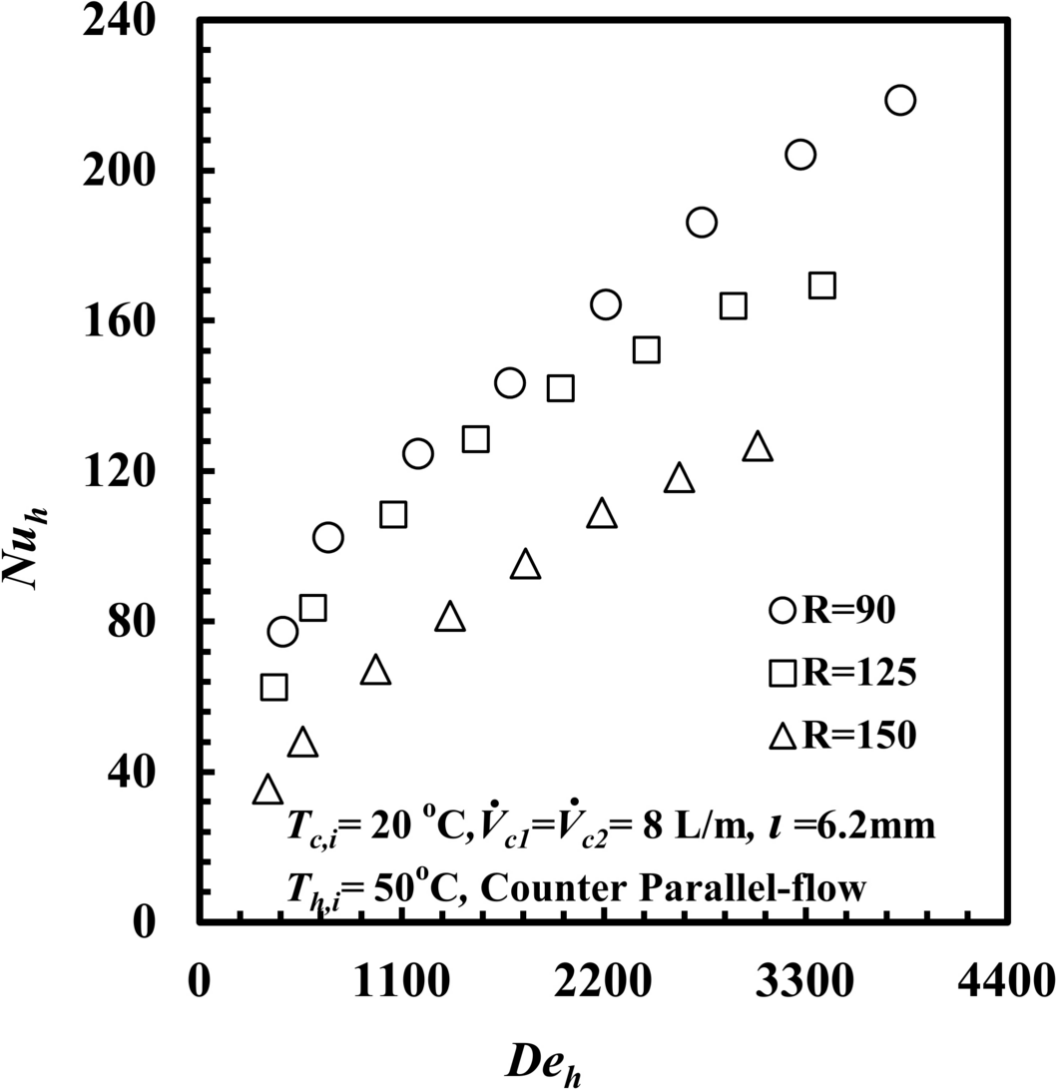
*Nu*_*h*_ versus *De*_*h*_ with different *THTHE* coil radius.

**Fig. 9:**
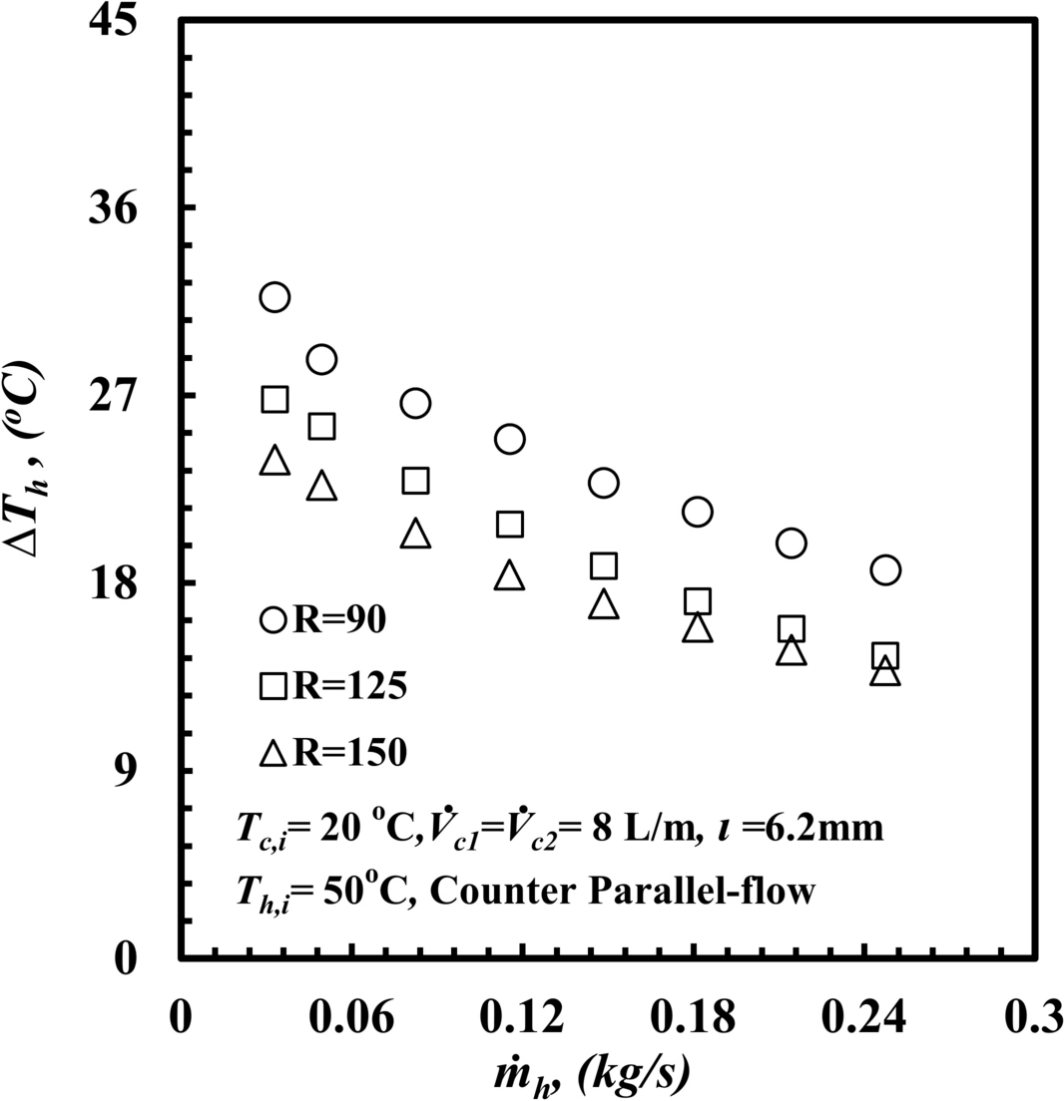
Hot temperature difference versus mass flow rate with different coil radius of *THTHE.*

**Fig. 10:**
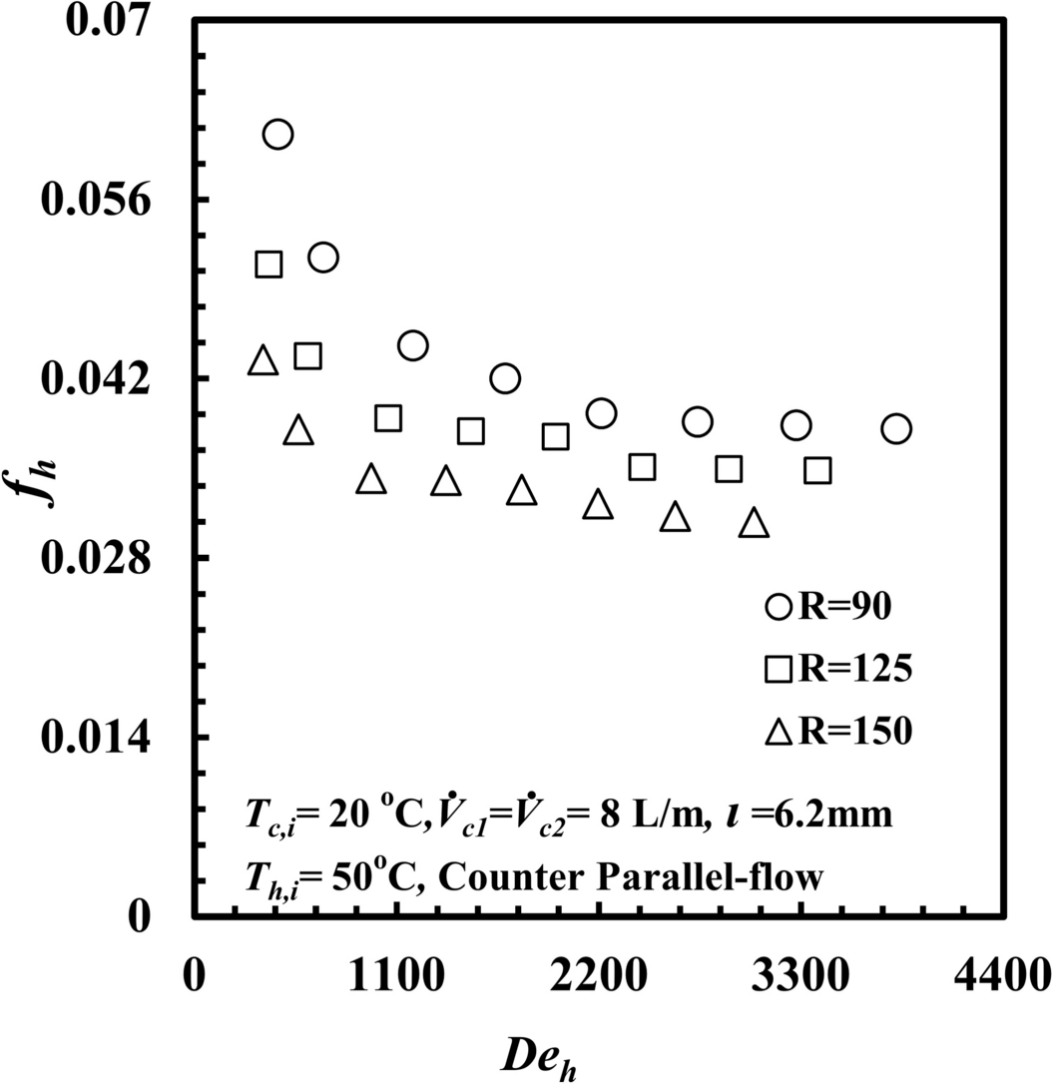
Friction factor versus Dean number with different coil radius of *THTHE*.

The effect of the coil radius on the thermal-hydraulic performance of a *THTHE* is the main point of interest. Three different coils with different coil radii, namely, specimen A with a coil radius of 150 mm, specimen B with a coil radius of 125 mm, and specimen C with a coil radius of 90 mm, are presented. The tests were carried out at *T*_*h,i*_ = 50 °C, with parallel-flow and cold-water flow rates of 8 L/m for both the inner and outer tubes. The effect of the coil radius on *Nu*_*h*_ against *De*_*h*_ is presented in Fig. 8. The *Nu*_*h*_ clearly increases with increasing *De*_*h*_ for all test samples. Moreover, the *Nu*_*h*_ for coil radius *R* = 90 mm provides the highest value compared with any other coil radius. At a particular value of *De*_*h*_ = 2860, the *Nu*_*h*_ for a coil radius, *R,* of 90 mm is higher than those of 125 mm and 150 mm by approximately 17.6% and 58.2%, respectively. This refers to the effect of the curvature ratio. When the coil radius decreases, the curvature ratio increases, and the generated centrifugal force and secondary flow increase. This leads to an increase in *∆T*_*h*_ (Fig. 9), and consequently, the heat transfer rate and *Nu*_*h*_ increase. The effect of the coil radius on *f*_*h*_ against *De*_*h*_ is presented in Fig. 10. Clearly, *f*_*h*_ decreases with increasing *De*_*h*_ in all the test samples. Notably, *f*_*h*_ for a coil radius of *R* = 90 mm provides the highest value compared with those of the other coil radii. At a particular *De*_*h*_ of 2860, the friction factor for a coil radius of 90 mm is higher than that of 125 mm and 150 mm by approximately 10.4% and 25.5%, respectively. Owing to the effect of the curvature ratio, when the coil radius decreases, the curvature ratio increases. This leads to an increase in the fluid turbulence and the *f*_*h*_, and consequently an increase pressure drop occured. When the coil diameter decrease the coil curvature ratio (*d*_*hy*_/*2R*) increase, as the curvature ratio increases the turbulence of fluid flow and the secondary flow intensity that created due to centrifugal force increases through the helical coil, this increase in secondary flow generation leads to increase the heat transfer rate and *Nu* force.

**Fig. 11:**
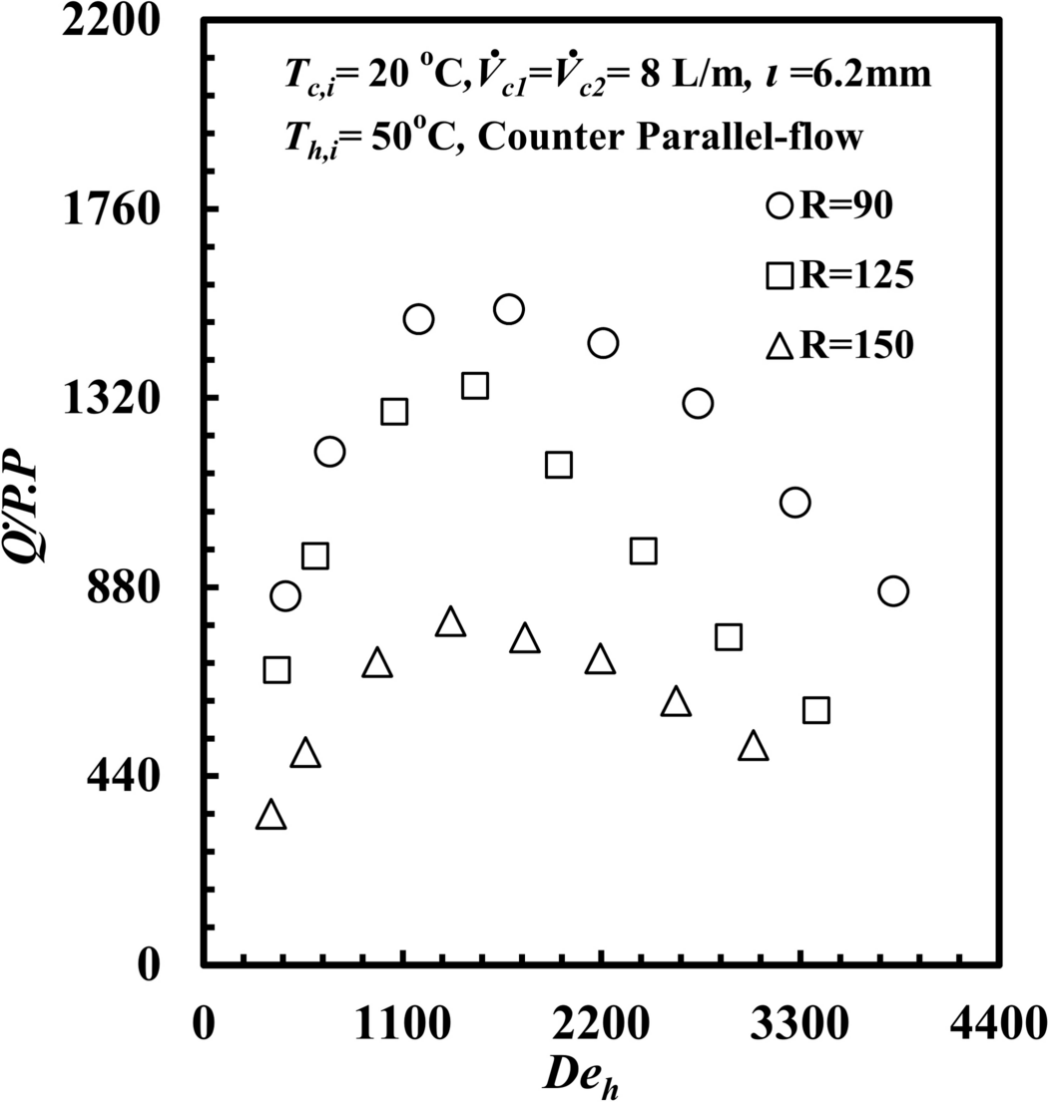
$\dot{Q}/PP$ versus *De*_*h*_ with different coil radius of *THTHE*.

**Fig. 12:**
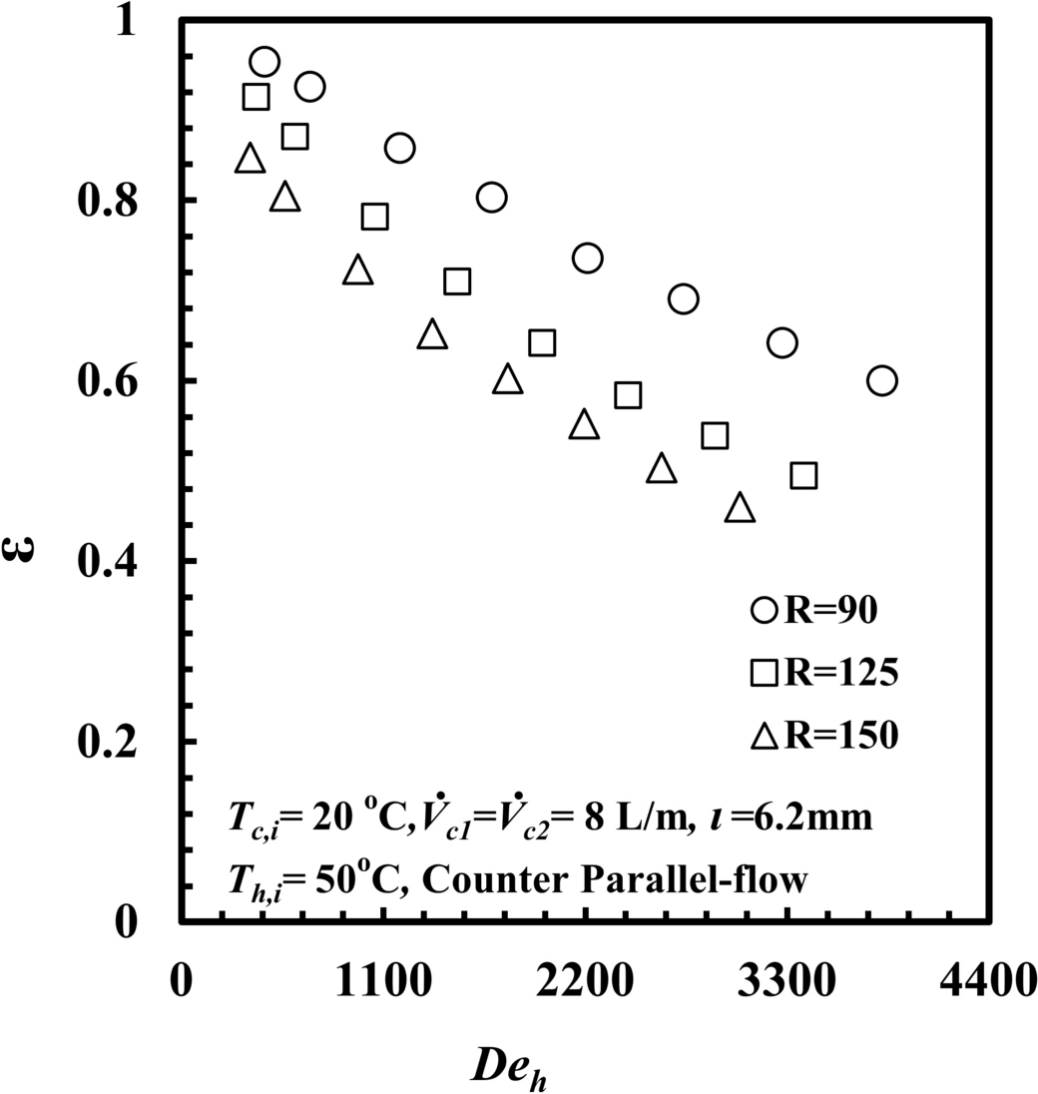
Effectiveness versus Dean number with different coil radius of *THTHE*.

The impact of coil radius variation on the heat transfer rate per pumping power, $\dot{Q}/PP$ and the Dean number, *De*_*h,*_ are displayed in Fig. 11, and it can be noted that $\dot{Q}/PP$ decreases as *De*_*h*_ decreases at all coil radii. It can also be seen that $\dot{Q}/PP$ for the coil radius *R* = 90 mm provides the highest value among the other coil radii. At a given Dean number, *De*_*h*_ of 2860, $\dot{Q}/PP$ for a coil radius of 90 mm is greater than that for a coil radius of 125 mm and 150 mm by approximately 58.8% and 160.6%, respectively. When the coil radius decreases, the curvature ratio (d_h_/2R) increases, and the turbulence of water increases, consequently increasing $\dot{Q}_{h}$ and the increase in $\dot{Q}/PP$ occurred. According to (Eq. 17), Fig. 12 shows the impact of the coil radius on the *THTHE, ε,* versus the Dean number, *De*_*h*_. The *ε* of *the THTHE* decreases with increasing *De*_*h*_ for all different coil radii. The figure shows that *ε* for a coil radius of *R* = 90 mm is greater than that for a coil radius of *R* = 125 mm and a coil radius of *R* = 150 mm. At a specific Dean number, *De*_*h*_ of 2860, the effectiveness, *ε*, for a coil radius of *R* = 90 mm is higher than that of 125 mm and 150 mm by approximately 23.6% and 41.7%, respectively. This can be attributed to the effect of the curvature ratio; when the coil radius decreases, the curvature ratio increases, and the turbulence of water increases, consequently increasing *∆T*_*h*_ at a coil radius of *R* = 90 mm compared with other coil radii. This leads to an increase in $\dot{Q}_{h}$ relative to *Q*_*max*_ and consequently an increase in the *ε*.

### Effect of inner annulus spacing

**Fig. 13:**
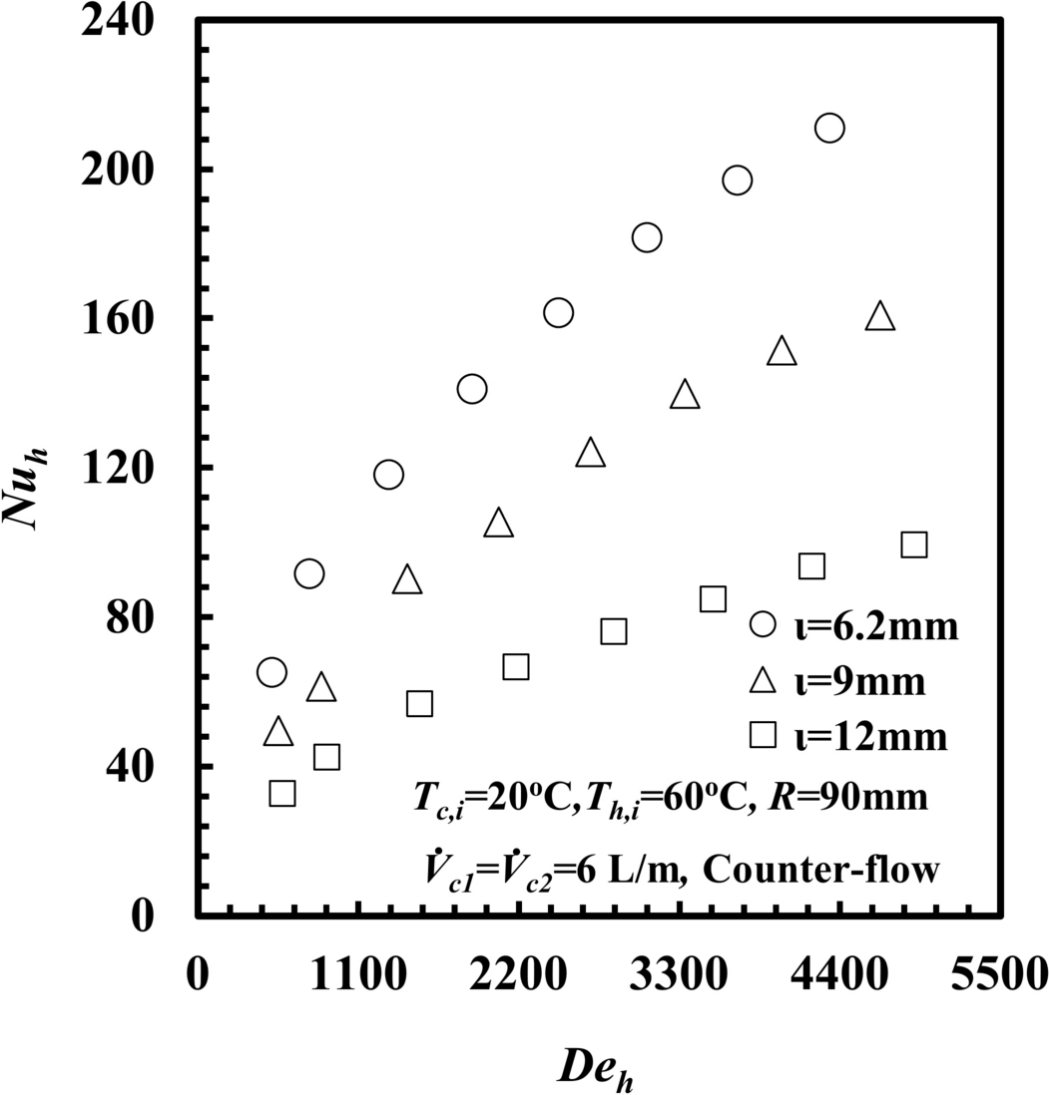
*Nu*_*h*_ versus *De*_*h*_ ahn number with different inner annulus spacing of *THTHE*.

The effect of the inner annulus spacing, $\iota$ is the main point of interest in this study. To verify the effect of $\iota$ on the thermal and hydraulic performance of the *THTHE*. Three different test samples, namely, C with $\iota$ = 6.2 mm, D with $\iota$ = 9 mm, and E with $\iota$ = 12 mm, were tested. The results are obtained at *T*_*h,i*_ = 60°C, counter flow, and a cold-water flow rate of 6 L/s for both the inner and outer fluids. The effects of the inner annulus spacing, *ι,* on *Nu*_*h*_ are presented in Fig. 13. It can be observed that *Nu*_*h*_ increases as *De*_*h*_ increases at all different values of $\iota$. Notably, *Nu*_*h*_ for *ι* = 6.2 mm is greater than that of the other values of $\iota$. At a certain value of *De*_*h*_ = 3300, *Nu*_*h*_ for an inner annulus spacing, *ι* of 6.2 mm is greater than that of 9 mm and 12 mm by approximately 36.6% and 130.4%, respectively.

**Fig. 14:**
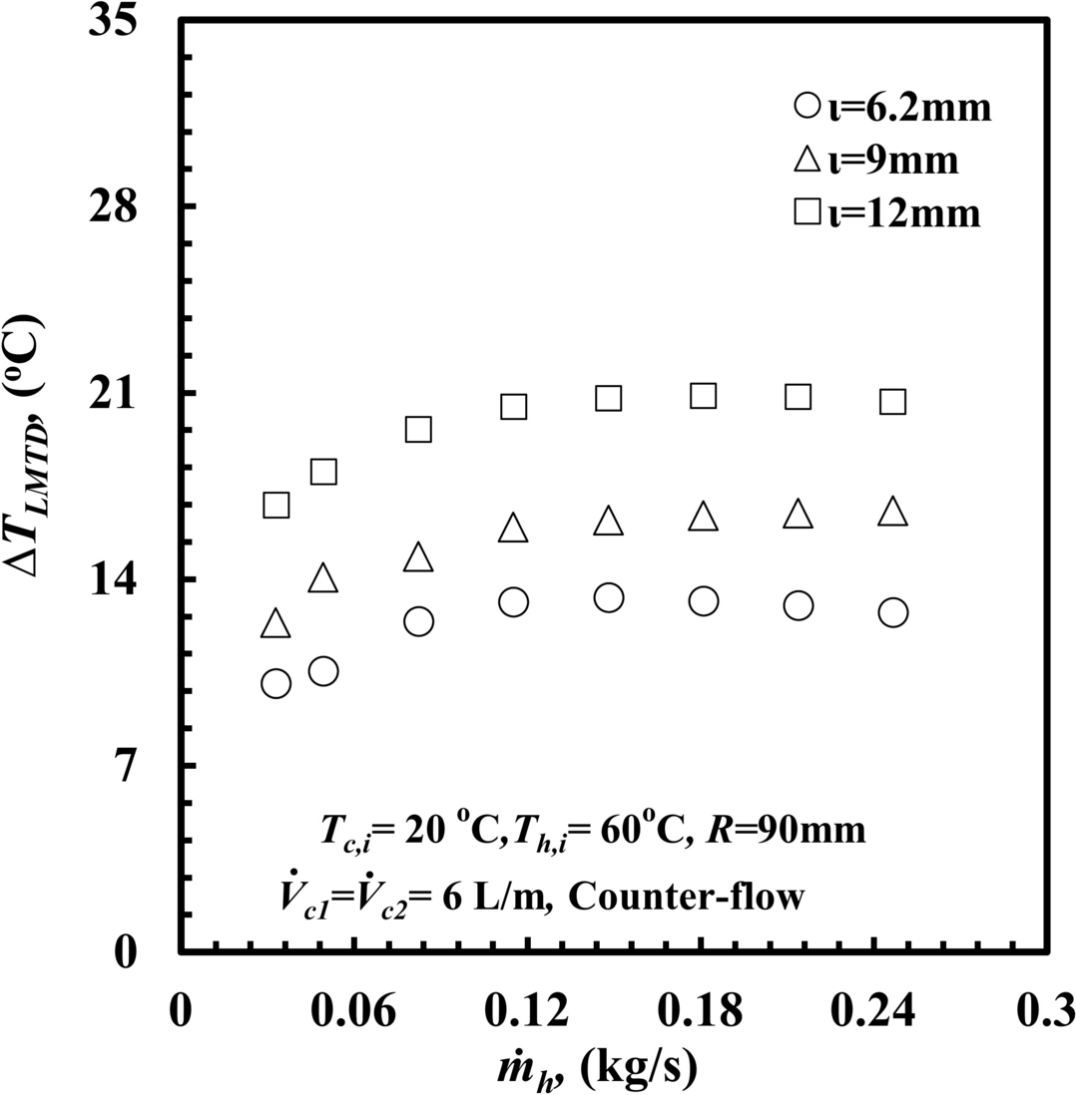
*ΔT*_*LMTD*_ versus $\dot{m}$_*h*_ with different inner annulus spacing of *THTHE*.

For the same coil diameter (2*R*), when $\iota$ decreases, the fluid velocity increases and leads to an increase in the heat transfer rate, $\dot{Q}_{h}$. On the other hand, the area of the inner surface, *A,* decreased, and the log mean temperature difference, *∆T*_*LMTD*_ (Fig. 14), decreased. However, the increase in $\dot{Q}_{h}$ is higher than the decreases in (*A*.*∆T*_*LMTD*_) product, which leads to an increase in *h* and consequently enhances *Nu*_*h*_ and vice versa.

Also, at the same coil diameter, (2*R*), when $\iota$ decreases the fluid velocity increased and the curvature ratio, (*d*_*hy*_*/*2*R*), decreased (this reflect to decrease the turbulence, secondary flow intensity). But the increase in the heat transfer duo to increasing the fluid velocity cover the reduction in heat transfer duo to curvature ratio, this lead also to increase $\dot{Q}_{h}$.

**Fig. 15:**
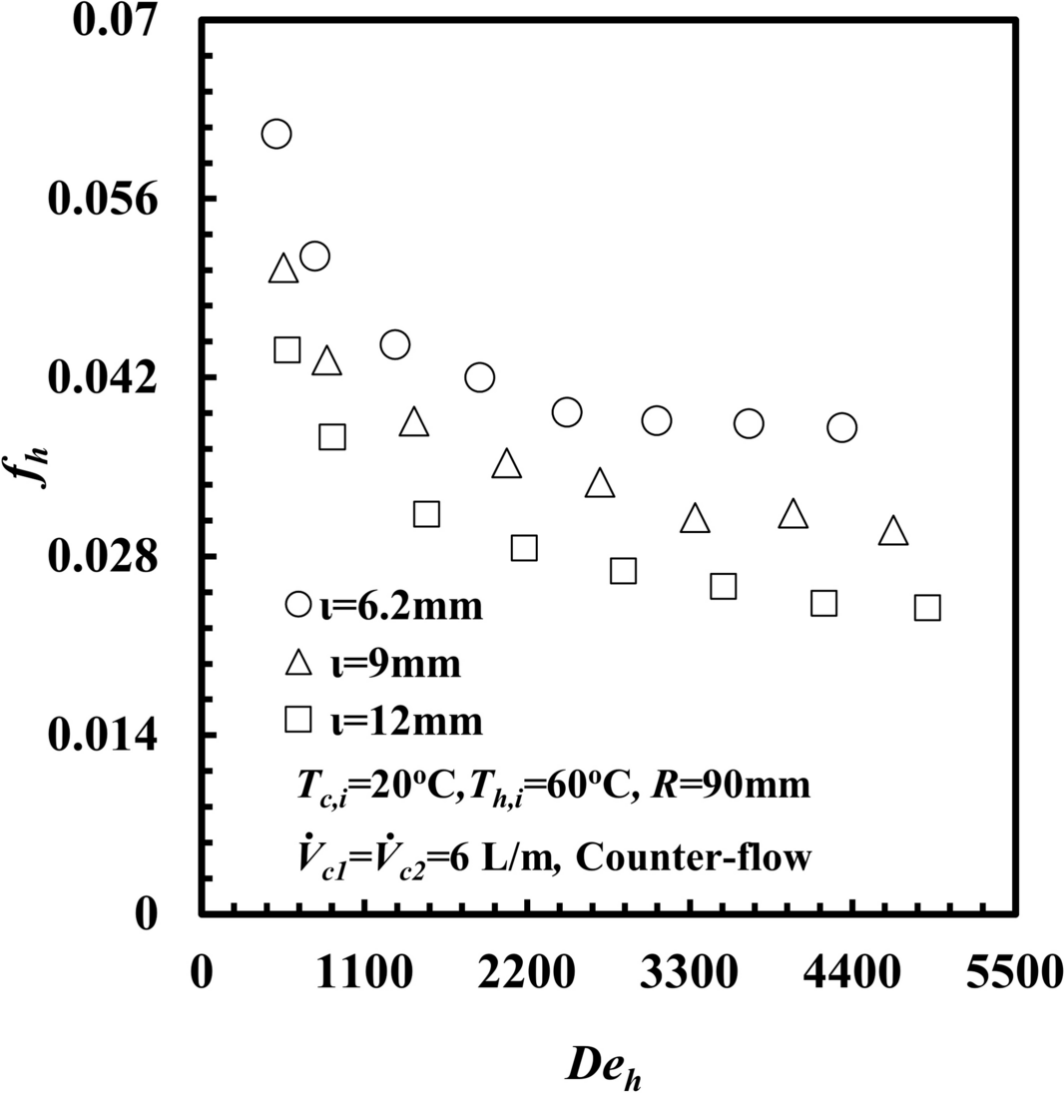
Friction factor versus Dean number with different inner annulus spacing of *THTHE*.

The effect of *ι* on *f*_*h*_ against *De*_*h*_ is presented in Fig. 15. It can be clearly seen that *f*_*h*_ decreases with increasing *De*_*h*_ for all test samples. Notably, the *f*_*h*_ for the inner annulus spacing of *d*_*hy*_ = 6.2 mm provides the highest value compared with that of the other *ι*. At a particular *De*_*h*_ of 3300, the *f*_*h*_ for *ι* = 6.2 mm is higher than that of 9 mm and 12 mm by approximately 27.3% and 47.4%, respectively. This can be attributed to Eq. (15). When the inner annulus spacing decreases, the hydraulic diameter decreases, and the pressure drop, *∆P,* in the inner annulus increases. On the other hand, the water square velocity, *v*^2^, increases, but the increase in *∆P* is greater than the increase in *v*^2^*,* which leads to an increasing friction factor, *f*_*h*_.

**Fig. 16:**
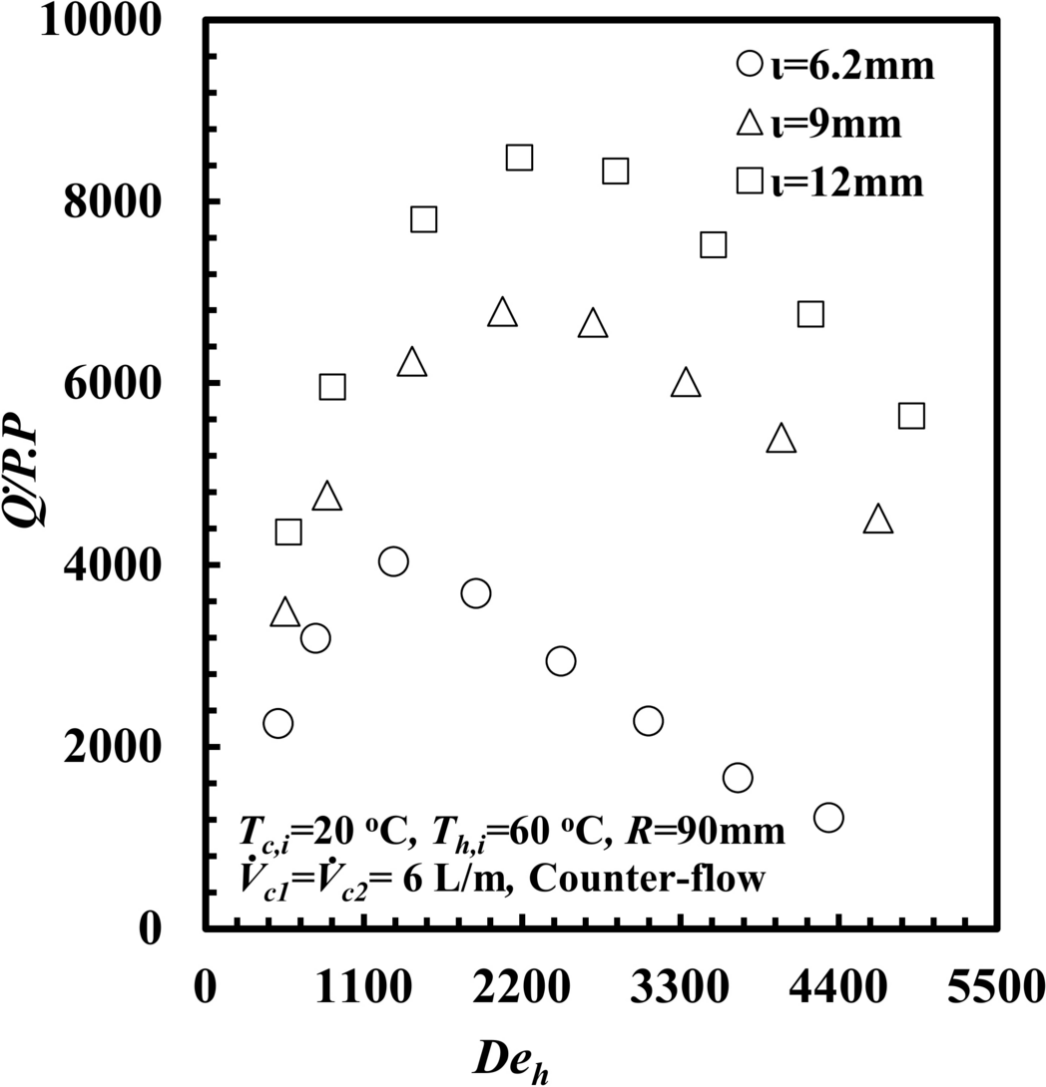
$\dot{Q}/PP$ versus *De*_*h*_ with different *THTHE* inner annulus spacing.

Influence of *ι* on $\dot{Q}/PP$ against *De*_*h*_ is shown in Fig. 16. It can be concluded that $\dot{Q}/PP$ decreases as *De*_*h*_ decreases for all different *ι* values, and the $\dot{Q}_{h}$ for *ι* = 12 mm supplies a higher $\dot{Q}/PP$ among others $\iota$. At *De*_*h*_ =3300, the $\dot{Q}/PP$ for *ι* = 12 mm is higher than that for 9 mm and 6.2 mm by approximately 30.1% and 286%, respectively.

Although decreasing *ι* enhances heat transfer in the helical coil, this enhancement comes at the expense of increasing the pressure drop and thus reduced $\dot{Q}/PP$ product values, and vice versa.

**Fig. 17:**
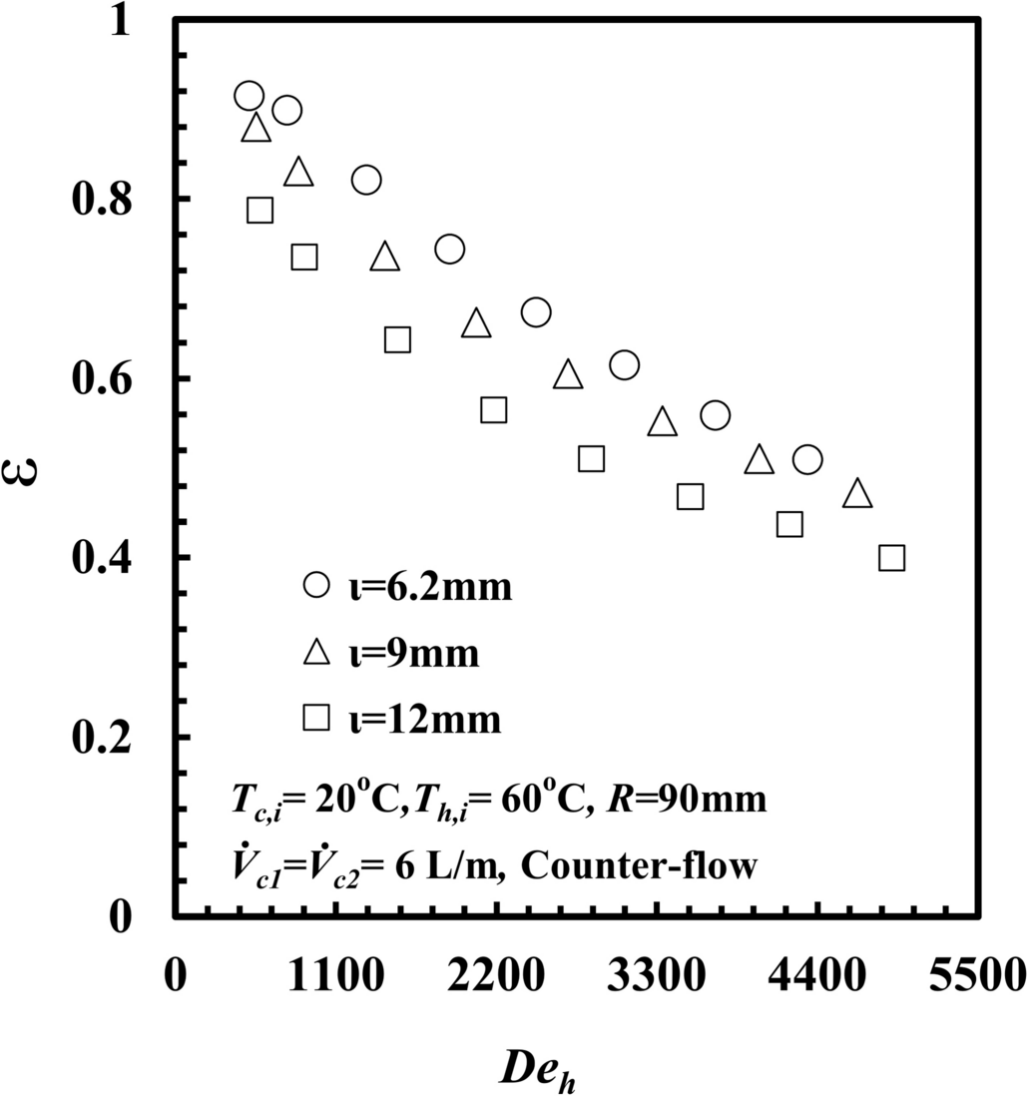
*ε* versus *De*_*h*_ with different *THTHE* inner annulus spacing.

When *ι* decreases, the fluid velocity and turbulence increases and the heat transfer rate, $\dot{Q}_{h}$ enhanced, at the expense of increasing the pressure drop and consequently the pumping power increases. therefore, the increase in the pumping power is greater than the increase in $\dot{Q}_{h}$, and consequently, *ι* = 6.2 mm results in a lower value of $\dot{Q}/PP$ Figure 17 shows the influence of *ι* on the *ε* of the *THTHE* versus *De*_*h*_. The figure shows that *ε* decreases with increasing *De*_*h*_ for all *ι*. The *ε* for *ι* = 6.2 mm is greater than that for the other *ι*. Similarly, for *De*_*h*_ = 3300, the effectiveness for *ι* = 6.2 mm is greater than that for *ι* = 9 mm and 12 mm by approximately 8% and 25%, respectively. This is due to the increase in *∆T*_*h*_ at *ι* = 6.2 mm compared with the other values of *ι*; hence, $\dot{Q}_{h}$ increased, increasing the effectiveness.

### Thermohydraulic performance criteria

**Fig. 18:**
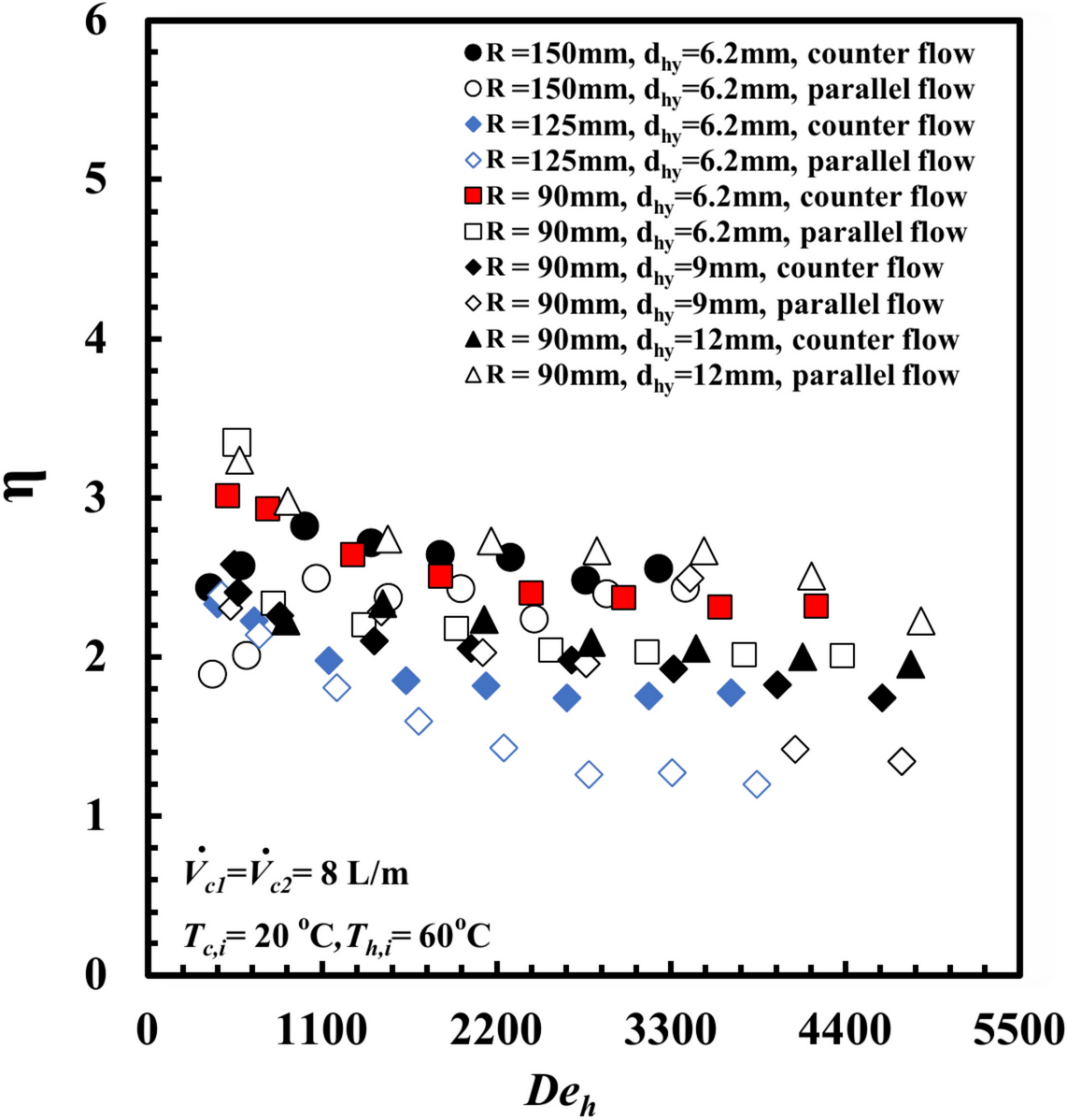
Performance index versus Dean number at different coil radius and inner annulus spacing.

The *THTHE* thermohydraulic performance criterion, *η,* is a point of interest. *η* indicates the extent of the increase in heat transfer, which leads to an increase in the *PP.* into the test samples. Figure 18 presents *η* against *Dn*_*h*_ at different *R* and *d*_*hy*_ values for both parallel and counter patterns and a *T*_*h,i*_ = 60 °C feed water temperature. It can be concluded that when *Dn*_*h*_ increases, *η* decreases. In addition, the maximum *η* value reached 3.3 for R = 90 mm at $\iota$ = 6.2 mm.

### Data correlations

**Fig. 19:**
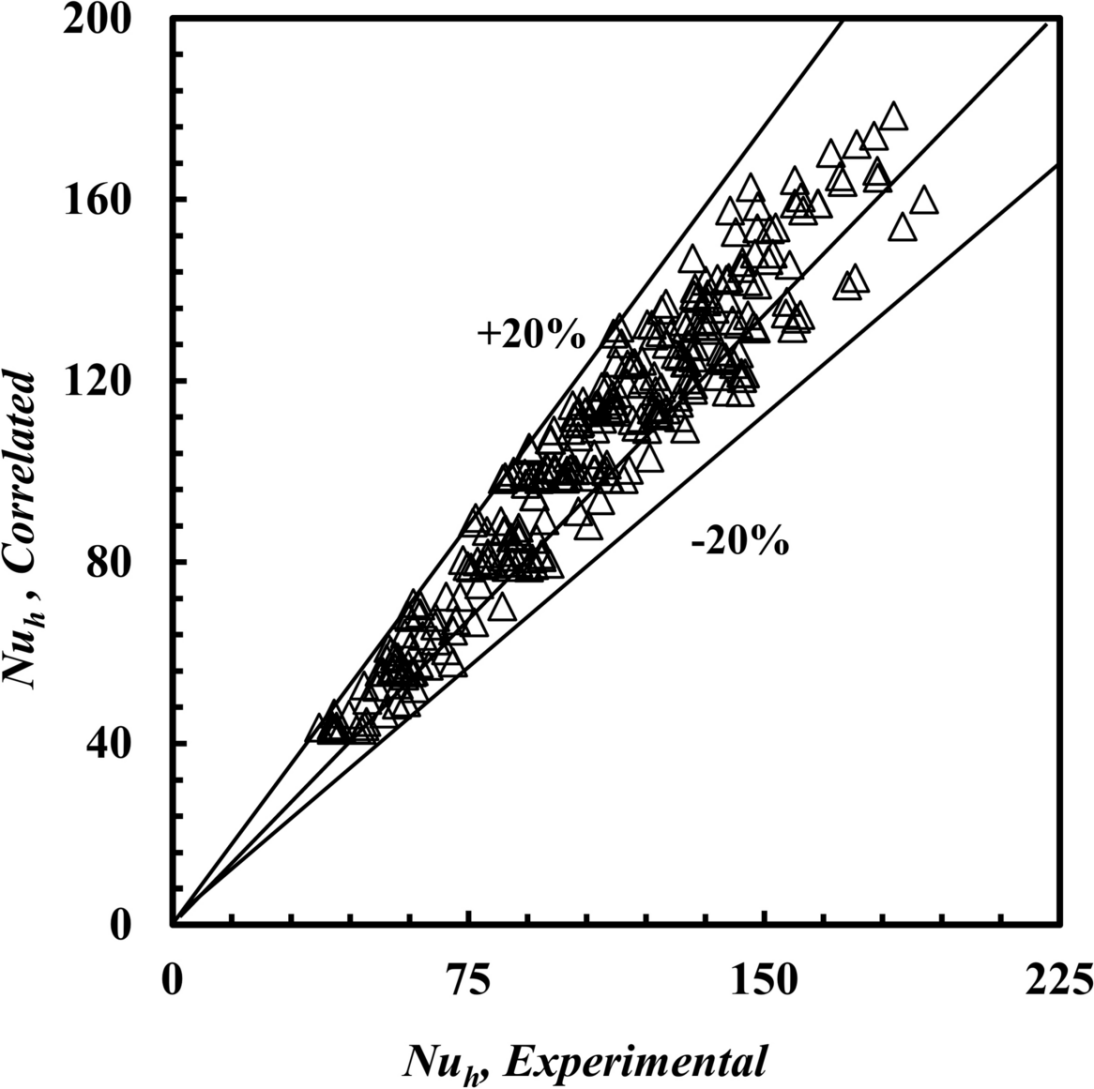
Confirmation of *Nu*_*h,*__*Correlation*_ and *Nu*_*h,*Experimental_.

**Fig. 20:**
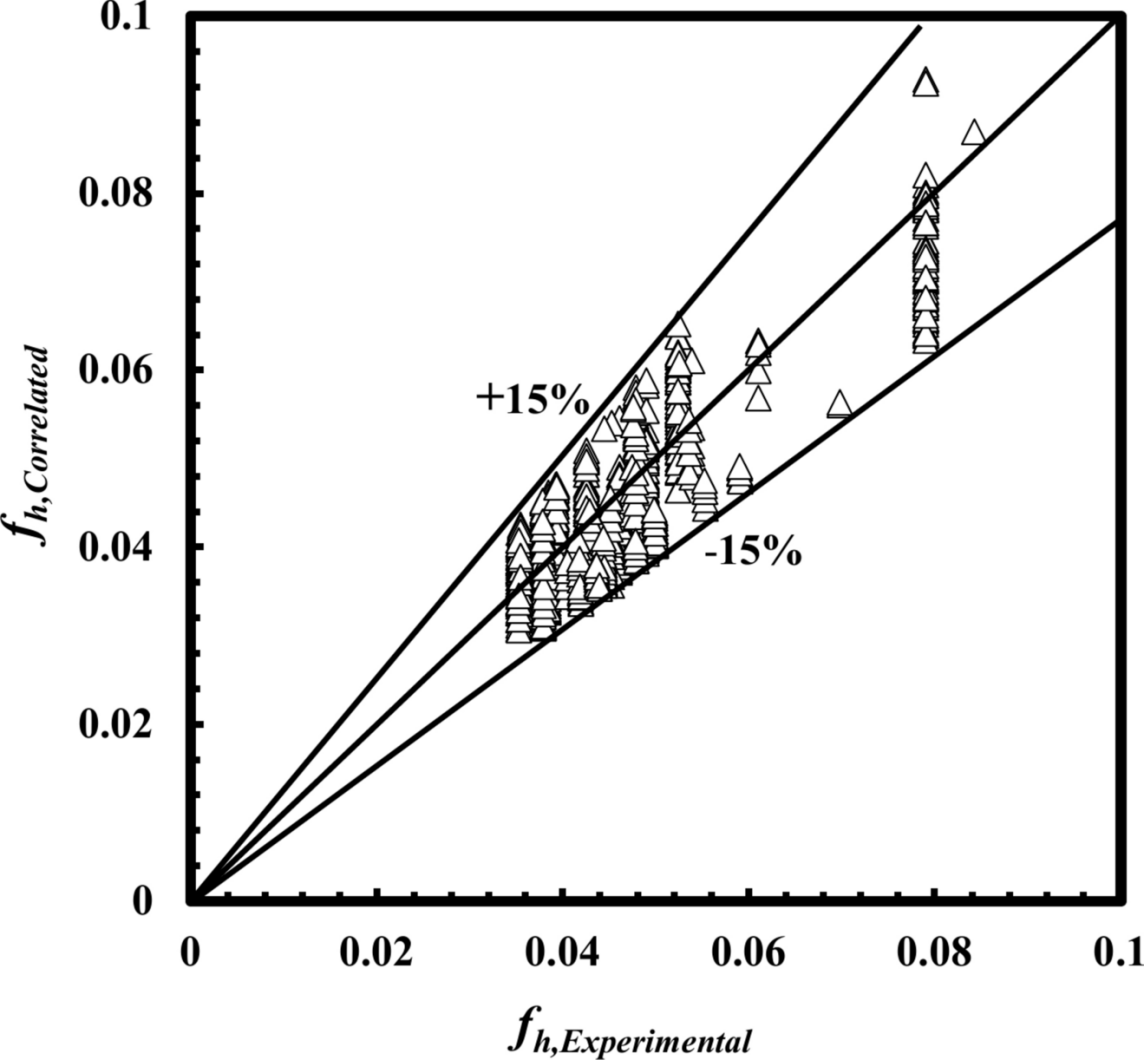
Confirmation of *f*_*h,*__*Correlation*_ and *f*_*h,*Experimental_.

**Fig. 21:**
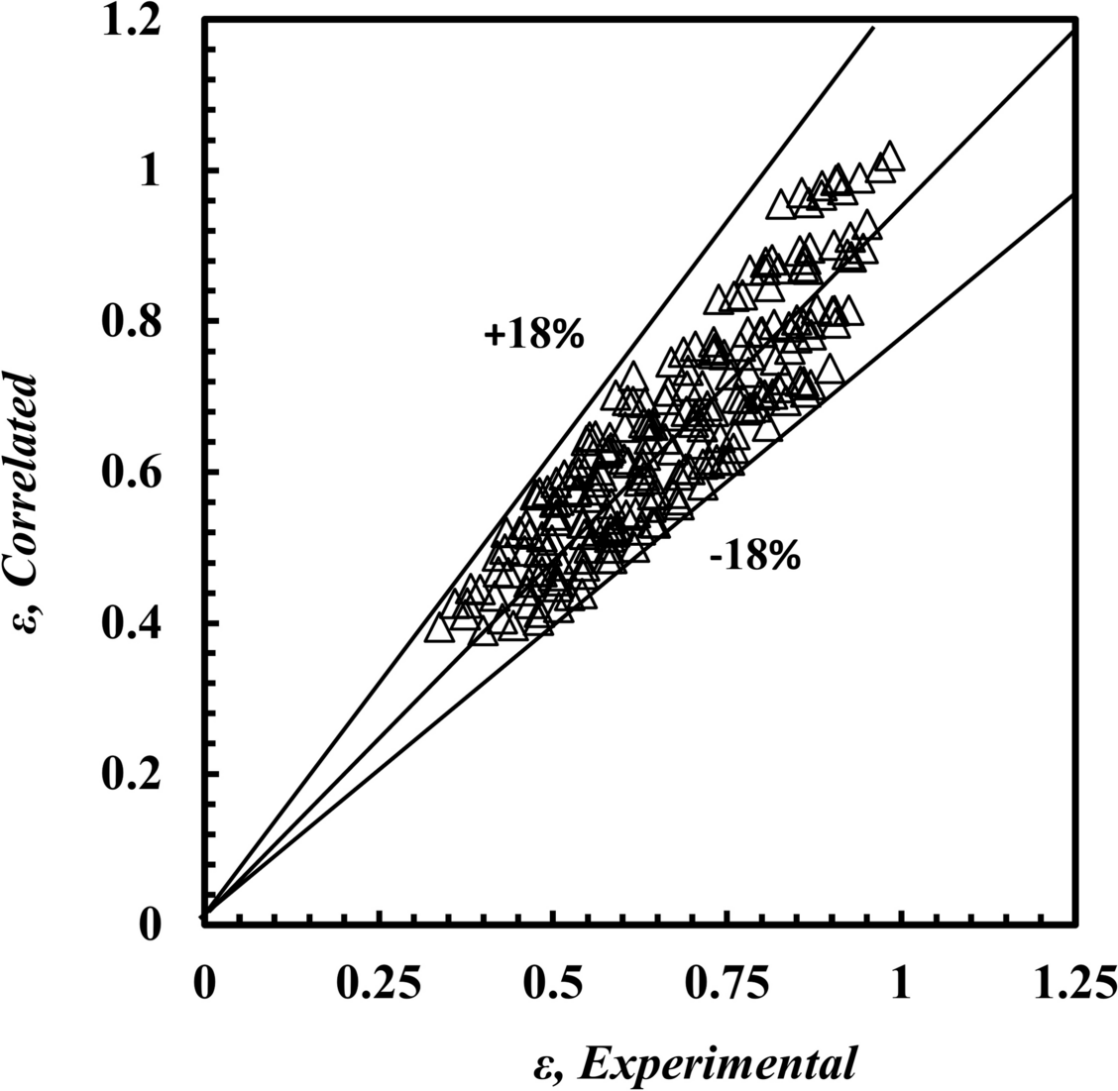
Confirmation of *ε* of *ε*,_*Correlation*_ and *ε*,_Experimental_.

In the present paper, three correlations are correlated according to a regression method for analysis for *Nu*_*h*_ Eq. (19), *f*_*h*_ Eq. (20), and the effectiveness of Eq. (21), as shown in Figs. 19, 20 and 21, respectively. The three expected correlations apply to ranges of 500 ≤ *De*_*h*_ ≤ 5500, 3.1 ≤ *Pr* ≤ 5.5, 50 °C ≤ *T*_*h*_*, *_*i*_ ≤ 80 °C as follows:

19 $$\begin{array}{*{20}c} {Nu_h = \, {0.569}De_{h}^{{{\mathbf{0}}.{\mathbf{487}}}} Pr_{h}^{{{\mathbf{1}}.{\mathbf{119}}}} } & {{\text{with a maximum deviation of }} \pm { 2}0\% } \\ \end{array}$$

20 $$\begin{array}{*{20}c} {f_{h} = \, {0.379}De_{h}^{{ - {\mathbf{0}}.{\mathbf{354}}}} Pr_{h}^{{{\mathbf{0}}.{\mathbf{452}}}} } & {{\text{with a maximum deviation of }} \pm { 15}\% } \\ \end{array}$$

21 $$\begin{array}{*{20}c} {\varepsilon = \, {0.828}De_{h}^{{ - {\mathbf{0}}.{\mathbf{203}}}} Pr_{h}^{{{\mathbf{0}}.{\mathbf{877}}}} } & {{\text{with a maximum deviation of }} \pm { 18}\% } \\ \end{array}$$

## Conclusions

A novel design of a *THTHE* characteristics were experiments carried out under the conditions of heat transfer from water to water. The design of *THTHE* is a modified design of *DHTHE*, to avoid the defects of *DHTHE* and to improve the heat transfer characteristics. In this study, five *THTHE* were designed, manufactured, and tested. The effects of *T*_*h,i*_, flow patterns, coil radius, hydraulic diameter, and the addition of *De* are the main points in this study. The main remarks were listed as:

- The *THTHE* Nusselt number, *Nu*_*h*_ was better than the *DHTHE* by 146.1% and 109.3%, respectively, for counter and parallel patterns.
- The experiments show that, *Nu*_*h*_ of a *THTHE* at *T*_*h,i*_ = 50°C is higher than that of 60°C, 70°C, and 80°C by approximately 10.5%, 30.5%, and 60.6%, respectively.
- The *Nu*_*h*_ increased in counter flow pattern predicts higher values among parallel, counter-parallel, and parallel-counter patterns by approximately 8.9%, 14.2%, and 20%, respectively, at the same conditions.
- The coil radius of 90 mm achieved higher *Nu*_*h*_ compared to that of 125 mm and 150 mm by approximately 17.6%, and 58.2%, respectively, at the expense of increasing friction factor by 10.4%, and 25.5%, respectively.
- The $\dot{Q}/PP$ for *THTHE* of* R* = 90 mm is higher than that of 125 mm, and 150 mm by approximately 58.8%, and 160.6%, respectively.
- At $\iota$ = 6.2 mm, the *Nu*_*h*_ achieved higher value compared to that of 9 mm and 12 mm by approximately 36.6%, and 130.4%, respectively, on chance of increasing *f* by 27.3%, and 47.4%, respectively.
- The effectiveness of the inner annulus spacing of 6.2 mm is greater than that of 9 mm and 12 mm by approximately 8% and 25%, respectively.
- New correlations to predict *Nu*_*h*_, *f*_*h*_ and *ε* were correlated.

### Future scope and recommendations

The triple tube heat exchanger needs an extra studies to investigate various designs of curved shapes as well as employing the nanofluids as a working fluids.

## Data Availability

Data availability statements The datasets generated during and/or analyzed during the current study are available from the corresponding author upon reasonable request.

## Abbreviations

*THTHE*: Triple helical tube heat exchanger (–)
*DHTHE*: Double helical tube heat exchanger (–)

## List of symbols

*A*: Area (m^2^)
*b*: Pitch (m)
*C*: Specific heat (kJ/kg.k)
*d*: Tube diameter (m)
*De*: Dean number (Re.δ^0.5^)(–)
*d*_*hy*_: Hydraulic diameter(m)
*f*: Friction factor (–)
*h*: Convective coefficient of heat transfer (W/m^2^.°C)
*k*: Conductivity (W/m.°C)
*L*: Length (m)
*LMTD*: Logarithmic-mean temperature difference (°C)
$\dot{m}$: Watfluider mass flow rate (kg/s)
*Nu*: Nusselt number (h.d_hy_/k) (–)
*N*: Number of turns (–)
*ΔP*: Pressure drop (pa)
*PP*: Pumping power (W)
*Pr*: Prandtl number (µ.cp/k) (–)
$\dot{Q}$˙: Heat transfer rate (W)
*Re*: Reynolds number (ρvd_hy_/µ) (–)
*R*: Radius (m)
*T *: Temperature (°C)

## Greek symbols

*v*: Velocity (m/s)
$\dot{V}$˙: Volumetric flow rate (L/m)
*δ*: Curvature ratio (*d*_*hy*_*/2R*) (–)
*ε*: Heat exchanger effectiveness (–)
*η*: Thermohydraulic performance index (–)
*ι*: Inner annulus spacing (m)
*µ*: Viscosity (kg/m.s)
*ρ*: Density (kg/m^3^)

## Subscripts

*avg*: Average (–)
*c*_1_: Inner tube fluid (–)
*c*_2_: Outer annulus fluid (–)
*h*: Inner annulus fluid (–)
*i*: Inner/Inlet (–)
*im*: Intermediate (–)
*max*: Maximum (–)
*min*: Minimum (–)
*o*: Outer tube/side (–)
